# Highlights of ophthalmological manifestations in newly diagnosed acute leukemia: a correlation with hematological parameters

**DOI:** 10.1007/s00277-024-05861-2

**Published:** 2024-07-10

**Authors:** Dina N. Laimon, Doaa H. Sakr, Basma Atef, Yasmine Shaaban

**Affiliations:** 1 Mansoura Ophthalmology Center, Faculty of Medicine, Mansoura University, Mansoura, Egypt; 2 Medical Oncology, Oncology Center Mansoura University, Faculty of Medicine, Mansoura University, Mansoura, Egypt; 3 Hematology Unit, Internal Medicine Department, Oncology Center, Faculty of Medicine, Mansoura University, Mansoura, Egypt

**Keywords:** ALL, Retinal hemorrhage, Roth spots, OS

## Abstract

Acute leukemia is a hematological malignancy affecting different organ systems including the eye and orbit through direct infiltration of tissues or secondary to hematological abnormalities. Ophthalmological manifestations in acute leukemia are variable ranging from asymptomatic presentation to serious manifestations that can alter the disease course and treatment. The purpose of this study is to detect the incidence of different ophthalmological manifestations in newly diagnosed acute leukemia patients and to assess the relationship between ocular findings and hematological characteristics and the sequel of these neoplasms. A cross-sectional study with analytical components was conducted on 222 newly diagnosed acute myeloid and acute lymphoblastic leukemia patients who presented at Oncology Center Mansoura University (OCMU) between January 2022 and February 2023. All patients underwent a complete ophthalmic evaluation at Mansoura Ophthalmology Center (MOC). The mean age was 43.45 ± 17.35 years (range, 17–85), and M/F was 137 (61.7%)/85 (38.3%). One-hundred and forty-four (64.9%) had acute myeloid leukemia (AML), and 78 (35.1%) had acute lymphoblastic leukemia (ALL). Ophthalmic manifestations were detected in 96 patients (43.2%). Among them, 4 (1.8%) had poor visual acuity. Retinal hemorrhage (19.8%) and Roth spots (17.1%) were the most common ocular manifestations. Other ophthalmological manifestations observed were orbital involvement (3.2%), ocular motility issues (1.4%), subconjunctival hemorrhage (5.9%), conjunctival chemosis (0.9%),lid swelling (4.1%), lid ecchymosis (3.2%), lagophthalmos (0.5%), lid ptosis (1.8%), retinal venous congestion & tortuosity (4.1%), preretinal hemorrhage (3.2%), vitreous hemorrhage (3.2%), macular affection (2.3%), retinal infiltration (1.8%), exudative retinal detachment (ERD) (1.8%), cotton-wool spots (0.9%), retinal vein occlusion (0.5%), papilledema (2.8%), optic disc infiltration (1.8%), disc pallor (1.8%).AML patients were significantly associated with a higher frequency of ocular affection, retinal hemorrhages, and Roth spots (***P*** 0.028, 0.003, and 0.046, respectively) compared to ALL patients. Retinal hemorrhage was statistically significantly associated with anemia (***P*** 0.021). Ophthalmological manifestations of acute leukemia are heterogeneous; they can be detected at initial presentations or relapse. Some manifestations are asymptomatic, others can affect visual acuity or even alter the disease course. Cooperation between ophthalmologists and haemato-oncologists is crucial for recognizing ocular involvement and disease management.

**Supplementary Information:**

The online version contains supplementary material available at 10.1007/s00277-024-05861-2.

## Introduction

Acute leukemia (AL) is a type of hematological cancer in which infiltrates of clonal, proliferative/poorly differentiated hematopoietic cells occupy the bone marrow, blood, and other tissues. Based on the origin of the abnormal hematopoietic cells involved, these disorders are classified into Acute Myeloid (AML) or Acute Lymphoblastic leukemia (ALL) [1].AML is predominantly a disease of older adults, with a median age at diagnosis of 68 years [2]. While 80% of ALL occurs in children, it represents a devastating disease when it occurs in adults [3]. The majority of newly diagnosed ALL cases have a precursor B-cell phenotype (B-ALL), and 12–15% have a precursor T-cell phenotype (T-ALL) [4].

Clinically evident ocular involvement is common in patients with leukemia and has been described in up to 50% of patients at the time of diagnosis [5]. It can compromise the functional and survival prognosis of the disease [6]. Different ophthalmological manifestations may occur due to either direct leukemic infiltration of different ocular tissues such as optic nerve, choroid, retina, iris, ciliary body, and anterior chamber or indirect owing to hematological abnormalities such as cytopenia and leukocytosis as well as secondary to chemotherapy or immunosuppression. Common indirect signs are retinal, pre-retinal, vitreous hemorrhages, infections, and retinal venous occlusions [7]. Other clinical signs comprise: Roth's spots, cotton wool spots, exudates, retinal venous tortuosity, perivascular sheathing, and neovascularization [8].

There have been relatively few reports focusing on the prevalence of ocular manifestations in newly diagnosed acute leukemia and its relation to the general features of the disease. As far as we know, this point has not been investigated before in the Delta region.

Hence, our study aims to highlight this unrecognized issue by evaluating ocular involvement in patients with ALL and AML attending Oncology Center Mansoura University at the time of diagnosis and correlating these findings to hematological parameters as a part of collaboration between Mansoura Oncology and Ophthalmology Centers.

## Patients and methods

### Study design

This is a cross-sectional study with an analytical component conducted on two-hundred and twenty-two newly diagnosed Acute Myeloid Leukemia (AML) and Acute Lymphoblastic Leukemia (ALL) patients who attended OCMU between January 2022 and February 2023.

### Sample size

(n) was calculated by the following formula (Daniel and Cross, 2018):$\textrm{n}=\frac{{\textrm{z}}^2\times \textrm{P}\times \left(1-\textrm{P}\right)}{{\textrm{d}}^2}$, a total sample size of 169 patients achieves 85% confidence level (*Z* = 1.44) for an expected prevalence of 28.4% (based on a previous study by Bukhari et al., 2021 [9] who reported a prevalence (P) of ocular manifestations of 28.4% in patients with acute leukemia) and an acceptable margin of error (d) of ± 5%.

To study the association between ocular manifestations and complete blood count (CBC) findings, a medium effect size (*d* = 0.6) is expected, based on the results of an earlier study by Dhasmana et al. (2016) [10]. For this study, a sample size of 45 patients with ocular manifestations and 45 patients without ocular manifestations is required. This sample size will result in a power of 80.37% to reject the null hypothesis of zero effect size when the population effect size is 0.60 and the significance level (α) is 0.050, using a two-sided two-sample equal-variance t-test [11].

### Study approval and data collection

The study was approved by the (Code Number: R.22.12.1989) Mansoura University Institutional Ethics Committee (IRB) guidelines in agreement with the Helsinki Declaration of 1975, revised in 2008. Newly diagnosed AML and ALL, either primary or secondary adult patients (≥18 years) of both genders were included in this study after obtaining their consent. We excluded Relapsed/Refractory acute leukemias and patients with pre-existing ocular disorders preceding the diagnosis of leukemia at the time of enrollment. Data was collected including the details of history, physical, ophthalmological examination, diagnostic workup, and therapy outcome from the electronic medical records of both the Oncology Center and Ophthalmology Centers in Mansoura University (Ibn Sina Hospital management system http://srv137.mans.edu.eg/mus/newSystem/).

The following clinical characteristics of AL patients at the time of diagnosis were tabulated: age, gender, laboratory investigations, including CBC with differential leucocytic counts, and blasts in peripheral blood, bone marrow (BM) examinations with cytogenetics, molecular analysis and immunophenotyping by flow cytometry (FCM) and cytochemistry. The status of central nervous system (CNS) involvement was investigated by conventional or flow cytometry analysis of the cerebrospinal fluid (CSF). Results of Brain CT/MRI (performed in case of neurologic or neuro-ophthalmic findings to exclude CNS hemorrhage or leukemic infiltration). Imaging of nasal and paranasal sinuses was also collected and recorded. The patients were classified using the European LeukemiaNet (2017) [12], and response assessment results for induction and salvage chemotherapies were documented.

Treatment protocols as per institutional guidelines are as follows:

1. Standard Intensive chemotherapy treatment (ICT) for AML: cytarabine-based with an anthracycline protocol for induction ('7+3') or high-dose cytarabine in consolidation or salvage protocols.
2. Low-intensity CT for AML: such as Hypomethylating agents (HMAs) e.g., azacitidine or low-dose cytarabine (LDAC).
3. Standard intensity CT for ALL: Hyper C-VAD protocol for patients ≥ 40 years old.
4. Pediatric-inspired ALL protocols: such as Augmented BFM, and GRAALL for adult and young adolescent patients.
5. Less intensified CT for ALL: such as Vincristine/corticosteroids for elderly or frail patients.
6. Best supportive care (BSC) was defined as cytoreductive therapy or no acute leukemia-specific treatment with blood product transfusion.
7. Salvage chemotherapy [FLAG or HAM] protocols for relapsed/refractory patients.
8. Tyrosine kinase inhibitors (TKI) were added for BCR-ABL1 positive acute leukemias.

### Ophthalmological assessment of the studied patients

Fundus examination is routinely performed for all newly diagnosed acute leukemia patients and whenever indicated in other events e.g., occurrence of neurological &/or ophthalmological symptoms and relapse. *Dr. Dina Laimon*; lecturer of ophthalmology at Mansoura Ophthalmology Center (MOC) performed a comprehensive examination for acute leukemia patients. From June 2022 till February 2023, she has conducted thorough examinations and has also reviewed previous ophthalmological reports from January to May 2022, and re-examined patients diagnosed in that period if necessary. The following parameters were included:

- Uncorrected distance visual acuity (UDVA) using Snellen chart.
- Detailed slit-lamp anterior segment examination.
- Fundus examination: using slit lamp biomicroscopy with non-contact Volk 90D lens.
- Assessment of ocular motility in all directions of gaze.
- Examination of ocular adnexa.
- Tonometry using Icare ONE, Finland Oy, Espoo, Finland).
- Ophthalmological imaging: using three-dimensional deep range imaging OCT Triton Plus (3D DRI OCT Triton (plus), Topcon Corporation, Tokyo, Japan) whenever indicated.

### Objectives of this study

The primary objective of the study was to assess the frequency of ophthalmological manifestations detected in newly diagnosed AML and ALL patients. The Secondary objective was to correlate these findings with hematological parameters at enrollment.

### Statistical analysis

The collected data was analyzed using the Statistical Package for Social Science (IBM Corp. Released 2017. IBM SPSS Statistics for Windows, Version 25.0. Armonk, NY: IBM Corp.). Kolmogorov Smirnov test was used to test the normality of data. For parametric data, mean and SD were used, while for non-parametric data, median, minimum, and maximum were used to describe the data. The chi-square test was used to examine the relationship between two qualitative variables. Fisher Exact or Monte Carlo tests were used to examine the relationship between two qualitative variables when the expected count is less than 5 in more than 20% of cells. Student T Test was used to assess the statistical significance of the difference of parametric variable between the two-study group means. The Mann-Whitney Test was used to assess the statistical significance of the difference of a non-parametric variable between two study groups. The ROC Curve (receiver operating characteristic) offers a useful way to evaluate the sensitivity and specificity of quantitative diagnostic measures that categorize cases into one of two groups. The optimum cut-off point was the one that maximized the AUC value. AUC is a test with an area greater than 0.9 that has high accuracy, while 0.7–0.9 indicates moderate accuracy, 0.5–0.7, low accuracy, and 0.5 a chance result. The log-rank test was used to evaluate the null hypothesis that there was no difference between the populations in the probability of an event (here a death) at any time point. A *p*-value is considered significant if <0.05 at a confidence interval of 95%.

## Results

### Clinical characteristics and treatment outcomes of the studied Acute leukemia patients

Newly diagnosed 222 AL patients (AML [*n*=144] and ALL [*n*=78]) were included at our center between January 2022 and February 2023. Their reported clinical and laboratory characteristics are listed in (Table 1). At presentation, the male and female percentages were 61.7% (*n*=137) and 38.3% (*n*=85) with a mean age of 43.45 ± 17.35 years (range, 17–85) among all cases. AML patients showed an older mean age of 46.7±17.6 *(P<0.001)*, while the ALL patients showed significant male predominance of 70.5% (*n*=55) *(P=0.047).* AML patients had significantly higher hemoglobin concentrations *(P=0.005),* lower platelet counts (*P=0.029*), as well as lower peripheral blood, and bone marrow blast percentages *(P<0.001*) compared to ALL patients.

**Table 1:** Clinical Characteristics and Clinical outcomes of Acute leukemia patients

	All Cohort *N* = 222	AML *N* = 144	ALL *N* = 78	p
Gender				
Male	137 (61.7%)	82 (56.9%)	55 (70.5%)	0.047*
Female	85 (38.3%)	62 (43.1%)	23 (29.5%)	
Age				
Mean ± SD.	43.45 ± 17.35	46.7±17.6	37.5±15.3	<0.001*
Type of induction treatment				
BSC	20 (9.0%)			
Standard intensity for AML	105 (47.3%)	105 (47.3%)		
Low intensity for AML	26 (11.7%)	26 (11.7%)		
Standard intensity ALL	32 (14.4%)		32 (14.4%)	
Pediatric inspired ALL	34 (15.3%)		34 (15.3%)	
Low intensity ALL	5 (2.3%)		5 (2.3%)	
Hb (g/dl)				
Mean ± SD.	8.52 ± 2.05	8.23 ± 1.91	9.04 ± 2.21	0.005*
TLC (k/uL)				
Median (Min. – Max.)	19 (0.3 – 670)	17.5 (0.3 – 300)	25 (0.4 – 670)	0.610
Plt count (k/uL)				
Median (Min. – Max.)	32 (2 – 512)	29 (4.5 – 320)	47.5 (2 – 512)	0.029*
Blast % in PB				
Median (Min. – Max.)	60 (5 – 98)	55 (5 – 96)	76.5 (15 – 98)	<0.001*
Blast % in BM				
Mean ± SD.	74.82 ± 21.99	71.01 ± 22.96	81.85 ± 18.22	<0.001*
Cytogenetics				
Negative	158 (71.2%)	88 (61.1%)	70 (89.7%)	<0.001*
Positive	64 (28.8%)	56 (38.9%)	8 (10.3%)	
Molecular abnormality at diagnosis	19 (8.6%)	19 (13.2%)	0 (0.0%)	0.001*
FLT3-ITD	17(7.67%)	17(11.8%)	0 (0.0%)	
NPM1	2(0.9%)	2(1.39%)	0 (0.0%)	
BCR/ABL1	21 (9.5%)	2 (1.4%)	19 (24.4%)	<0.001*
Risk Stratification				
Favorable	71 (32.0%)	46 (31.9%)	25 (32.1%)	<0.001*
Intermediate	86 (38.7%)	69 (47.9%)	17 (21.8%)	
Poor	65 (29.3%)	29 (20.1%)	36 (46.2%)	
TKI therapy	21 (9.5%)	8 (5.6%)	13 (16.7%)	0.007*
Response to induction treatment				
CR	98 (44.1%)	53 (36.8%)	45 (57.7%)	0.001*
PR	7 (3.2%)	4 (2.8%)	3 (3.8%)	
Refractory	39 (17.6%)	24 (16.7%)	15 (19.2%)	
Induction death	35 (15.8%)	33 (22.9%)	2 (2.6%)	
Not applicable	43 (19.4%)	30 (20.8%)	13 (16.7%)	
Relapse after CR	26/98 (26.5%)	12/53 (22.6%)	14/45 (31.1%)	0.344
Type of salvage for R/R				
No salvage	175 (78.8%)	121 (84.0%)	54 (69.2%)	0.036*
HAM or FLAG (aggressive treatment)	39 (17.6%)	19 (13.2%)	20 (25.6%)	
Low intensity therapy	8 (3.6%)	4 (2.8%)	4 (5.1%)	
Response to salvage				
CR	18 (8.1%)	8 (5.6%)	10 (12.8%)	0.075
PR	22 (9.9%)	12 (8.3%)	10 (12.8%)	
Not applicable	182 (82.0%)	124 (86.1%)	58 (74.4%)	
CSF infiltration at diagnosis	14/154 (9.1%)	7/80 (8.8%)	7/74 (9.5%)	0.878
CSF infiltration at another event	11/85 (12.9%)	4/31 (12.9%)	7/54 (13%)	1.000
IT chemotherapy for CNS infiltration	28 (12.6%)	11 (7.6%)	17 (21.8%)	0.002*
Response to IT chemotherapy				
NR	5 (17.9%)	2 (18.2%)	3 (17.6%)	0.102
CR	16 (57.1%)	4 (36.4%)	12 (70.6%)	
Not applicable	7 (25.0%)	5 (45.5%)	2 (11.8%)	
Brain radiology	27/112 (24.1%)	17/65 (26.2%)	10/47 (21.3%)	0.551
Nasal and paranasal radiology	33/99 (33.3%)	23/66 (34.8%)	10/33 (30.3%)	0.651
Cranial irradiation	31 (14.0%)	2 (1.4%)	29 (37.2%)	<0.001*
SCT	6 (2.7%)	4 (2.8%)	2 (2.6%)	1.000

*SD.* standard deviation, *Min.* minimum, *Max.* maximum, *p* comparingthe different categories, *: significant, BSC: best supportive care, Hb: hemoglobin, TLC: total leucocytic count, Plt: platelet, TKI: tyrosine kinase inhibitor, CR: complete response, PR: partial response, R/R: relapsed/refractory, CSF: cerebrospinal fluid, it: intrathecal, CNS: central nervous system, NR: no response, SCT: stem cell transplant.

AML patients showed a significantly higher frequency of abnormal cytogenetics when compared to ALL patients (*P<0.001*). BCR/ABL1 was more significantly detected in 24.4% of ALL cases (*n*=19) *(p<0.001),* while other molecular abnormalities were detected among the AML group *(P=0.001).* As for the risk stratification among all the cohorts, 3 groups were identified: favorable [*n*=71(32.0%)], intermediate [*n*=86 (38.7%)], and poor [*n*=65 (29.3%)] with more AML patients in the intermediate and more ALL patients in poor risk groups *(P<0.001)* (Table 1).

Response assessment to induction treatment showed that 98 patients (44.1%) achieved complete remission (CR), 7 patients (3.2%) achieved partial remission (PR), and 39 patients (17.6%) were refractory. ALL cases achieved more CR and PR compared to AML cases [(57.7% *vs.* 36.8%) and (3.8% *vs.* 2.8%), respectively (*P=0.001*)]. Additionally, more ALL patients were refractory [19.2% *vs.* 16.7% AML patients (*P=0.001*)]. Unfortunately, 35 patients (15.8%) died during induction and 43 patients (19.4%) were not applicable for response evaluation.

Relapse was reported in (26.5%) patients with no significant difference between either AML or ALL arms (*P*=*0.344)*. Among the relapsed/refractory (R/R) AL patients, 39 (17.6%) received aggressive salvage chemotherapy, while 8 (3.6%) received low-intensity therapy because they were not fit for aggressive CT. Eighteen patients (8.1%) achieved CR and 22 patients (9.9%) achieved PR after receiving salvage therapy.

One hundred and fifty-four patients were evaluated for CNS infiltration by lumbar puncture and conventional or FCM analysis of CSF at diagnosis (patients with neurological symptoms at presentation or part of ALL workups); positive findings were detected in 14 patients (9.1%). Out of the 85 patients who were evaluated for CSF infiltration during other events, 11 patients (12.9%) were found to have infiltration. Data was available for 28 (12.6%) patients to evaluate the response to intrathecal (IT) chemotherapy given for CNS infiltration. Out of these 28 patients, 5 patients (17.9%) did not respond, 16 patients (57.1%) achieved CR, and 7 patients (25%) were not applicable for response evaluation (Table 1).

AML cases were significantly associated with a higher frequency of molecular abnormality at diagnosis, intermediate risk, and induction death. On the other hand, they had a lower frequency of BCR/ABL1, poor risk, TKI therapy, CR, and salvage chemotherapy, low-intensity therapy, PR, IT chemotherapy for CNS infiltration, and cranial irradiation when compared to ALL cases (Table 1).

The radiological data for brain, nasal, and paranasal sinuses are illustrated in (Supplementary Table 1).

### Ophthalmological manifestations and their relation to the studied acute leukemia patients

More than fifty percent of the cohort (56.8%) did not have any eye affection at diagnosis. Out of the patients who were examined, the numbers were distributed as follows: 11.7% had right involvement, 5.9% had left eye involvement, and 25.7% had bilateral involvement. In terms of visual acuity, 91.4% had good vision, while 6.8% had impaired vision and 1.8% had complete visual loss.

**Fig. 1:**
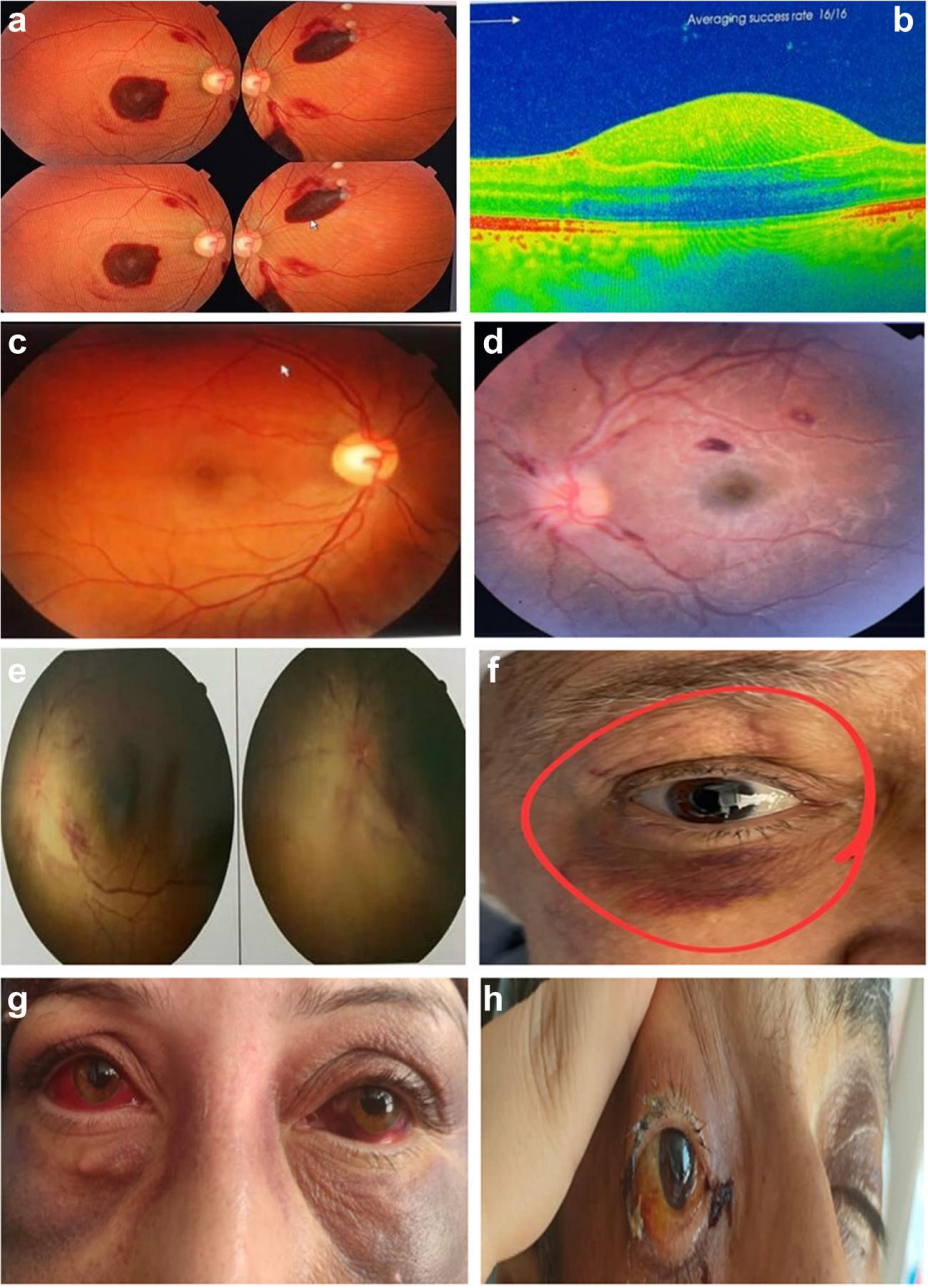
Some ophthalmological findings in the studied acute leukemia patients. **a**) Fundus photo of right and left eyes of AML patient: the right eye demonstrates the presence of sharply demarcated, dome-shaped epimacular hemorrhage in subhyaloid space anterior to internal limiting membrane (ILM) and scanned by OCT, while the left eye shows multiple areas of subhyaloid hemorrhage related to upper and lower temporal retinal vascular arcades. **b**) OCT of macula [OD] demonstrates large dome-shaped epimacular hemorrhage. **c**) Fundus photo of the right eye of the same patient two months after chemotherapy shows resolution of epimacular hemorrhage with subsequent macular hole. **d**) Fundus photo of the left eye of ALL patient demonstrating the presence of scattered and peripapillary flame-shaped hemorrhages and Roth spots which are fibrin-platelet plug at a site of vessel rupture. The photo also shows optic disc swelling at the nasal margin. **e**) Fundus photo of the left eye of AML patient with leukemic optic disc infiltration (disc swelling with obliteration of blood vessels surrounding the disc, obliterated cup, and disc hemorrhage with congested disc vessels). **f**) Right eye of AML patient with lower eyelid ecchymosis. **g**) AML patient with bilateral subconjunctival hemorrhage and bilateral lower lid ecchymosis. **h**) AML patient with right orbital cellulitis, proptosis, and conjunctival chemosis

Various ophthalmological findings were detected during the examination. These included lid ecchymosis (3.2%), lid ptosis (1.8%), lid swelling (4.1%), subconjunctival hemorrhage (5.9%), conjunctival chemosis (0.9%), preretinal hemorrhage (3.2%), retinal hemorrhage (19.8%), vitreous hemorrhage (3.2%), Roth spots (17.1%), cotton wool spots (0.9%), optic disc infiltration (1.8%), disc pallor (1.8%), papilledema (2.8%), venous congestion and tortuosity (4.1%), retinal infiltration (1.8%), retinal vein occlusion (0.5%), exudative retinal detachment (1.8%), ocular motility issues (1.4%), orbital involvement (3.2%), macula affection (2.3%), and lagophthalmos (0.5%). No keratopathy or iris involvement was detected (Table 2). Some of these findings are illustrated in (Fig. 1).

**Table 2:** Ophthalmological manifestations in newly diagnosed AML and ALL patients

	All Cohort *N* = 222	AML *N* = 144	ALL *N* = 78	*p*
Side for ophthalmological finding				
No eye affection	126 (56.8%)	72 (50.0%)	54 (69.2%)	0.028*
Right eye	26 (11.7%)	17 (11.8%)	9 (11.5%)	
Left eye	13 (5.9%)	10 (6.9%)	3 (3.8%)	
Bilateral	57 (25.7%)	45 (31.3%)	12 (15.4%)	
Visual acuity				
Good	203 (91.4%)	133 (92.4%)	70 (89.7%)	0.265
Impaired	15 (6.8%)	10 (6.9%)	5 (6.4%)	
Lost	4 (1.8%)	1 (0.7%)	3 (3.8%)	
Lid ecchymosis	7 (3.2%)	4 (2.8%)	3 (3.8%)	0.699
Lid ptosis	4 (1.8%)	1 (0.7%)	3 (3.8%)	0.126
Lid swelling	9 (4.1%)	7 (4.9%)	2 (2.6%)	0.499
Subconjunctival hemorrhage	13 (5.9%)	11 (7.6%)	2 (2.6%)	0.147
Conjunctival chemosis	2 (0.9%)	1 (0.7%)	1 (1.3%)	1.000
Preretinal hemorrhage	7 (3.2%)	5 (3.5%)	2 (2.6%)	1.000
Retinal hemorrhage	44 (19.8%)	37 (25.7%)	7 (9.0%)	0.003*
Vitreous hemorrhage	7 (3.2%)	7 (4.9%)	0 (0.0%)	0.099
Roth spots	38 (17.1%)	30 (20.8%)	8 (10.3%)	0.046*
Cotton wool spots	2 (0.9%)	2 (1.4%)	0 (0.0%)	0.542
Optic disc infiltration	4 (1.8%)	1 (0.7%)	3 (3.8%)	0.126
Optic disc pallor	4 (1.8%)	2 (1.4%)	2 (2.6%)	0.614
Papilledema	6 (2.8%)	3 (2.1%)	3 (3.8%)	0.426
Venous congestion & tortuosity	9 (4.1%)	4 (2.8%)	5 (6.4%)	0.284
Retinal infiltration	4 (1.8%)	1 (0.7%)	3 (3.8%)	0.126
Retinal vein occlusion	1 (0.5%)	1 (0.7%)	0 (0.0%)	1.000
Exudative retinal detachment	4 (1.8%)	4 (2.8%)	0 (0.0%)	0.300
Ocular motility	3 (1.4%)	1 (0.7%)	2 (2.6%)	0.283
Orbital involvement	7 (3.2%)	4 (2.8%)	3 (3.8%)	0.699
Macula affection	5 (2.3%)	5 (3.5%)	0 (0.0%)	0.165
Lagophthalmos	1 (0.5%)	1 (0.7%)	0 (0.0%)	1.000

p: comparing the different categories, *: significant

Compared to ALL patients, AML patients had a significantly higher frequency of only left or bilateral eye affection *(P=0.028)*, retinal hemorrhage *(P=0.003),* and Roth spots *(P=0.046)* (Table 2).

We studied the correlation between ophthalmological manifestations and different hematologic or leukemic parameters (Supplementary Tables 2-6). We found that CNS infiltration had a significant association with lid ecchymosis *(P=0.045).* The lower BM blast percentage was significantly associated with lid ptosis (*P=0.04*). Retinal hemorrhage was significantly associated with lower Hb concentration (7.88g/dL ± 1.96, *P* 0.021), higher incidence of intermediate and poor risk *(P=0.002),* and lower incidence of CNS infiltration *(P=0.021).* Retinal infiltration and exudative retinal detachment were significantly associated with higher BM blast percentage *(P=0.006 and 0.001,* respectively) (Table 3). However, we did not find any significant association between conjunctival chemosis, Roth spots, cotton wool spots, or papilledema with different hematologic or leukemic parameters. Our study also concludes that there is no significant association between leukemic CNS infiltration and optic disc or retinal infiltration and papilledema *(P>0.05).* Furthermore, ophthalmological abnormalities were not associated with response to induction chemotherapy, hematological relapse, or response to salvage chemotherapy (Supplementary Tables 2-6).

**Table 3:** Significant associations between ophthalmological findings and hematological parameters

	Visual acuity			Lid ecchymosis			Lid ptosis			Lid swelling		
	Good *N* = 203	impaired+ lost *N* = 19	*p*	Absent *N* = 215	Present *N* = 7	*p*	Absent *N* = 218	Present *N* = 4	*p*	Absent *N* = 213	Present *N* = 9	*p*
Blast % in BM												
Mean ± SD.	75 ± 21.7	72.8 ± 25.9	0.683	75.1 ± 21.9	67 ± 26.9	0.340	75.2 ± 21.7	52.5 ± 28.7	0.040^*^	75 ± 21.9	71.3 ± 25.3	0.628
CNS infiltration	24 (11.8%)	4 (21.1%)	0.273	25 (11.6%)	3 (42.9%)	0.045^*^	27 (12.4%)	1 (25.0%)	0.419	25 (11.7%)	3 (33.3%)	0.090
	Subconjunctival hemorrhage			Preretinal hemorrhage			Retinal hemorrhage			Vitreous hemorrhage		
	Absent *N* = 209	Present *N* = 13	*p*	Absent *N* = 215	Present *N* = 7	*p*	Absent *N* = 178	Present *N* = 44	*p*	Absent *N* = 215	Present *N* = 7	*p*
Hb (g/dl)												
Mean ± SD.	8.5 ± 2.06	8.78 ± 2.1	0.628	8.5 ± 2.08	9.07 ± 1.09	0.468	8.67 ± 2.05	7.88 ± 1.96	0.021^*^	8.54 ± 2.07	7.86 ± 1.36	0.390
Risk Stratification												
Favorable	65 (31.1%)	6 (46.2%)	0.467	70 (32.6%)	1 (14.3%)	0.567	61 (34.3%)	10 (22.7%)	0.002^*^	68 (31.6%)	3 (42.9%)	0.719
Intermediate	81 (38.8%)	5 (38.5%)		83 (38.6%)	3 (42.9%)		59 (33.1%)	27 (61.4%)		83 (38.6%)	3 (42.9%)	
Poor	63 (30.1%)	2 (15.4%)		62 (28.8%)	3 (42.9%)		58 (32.6%)	7 (15.9%)		64 (29.8%)	1 (14.3%)	
PR	20 (9.6%)	2 (15.4%)		22 (10.2%)	0 (0.0%)		21 (11.8%)	1 (2.3%)		21 (9.8%)	1 (14.3%)	
Not applicable	171 (81.8%)	11 (84.6%)		176 (81.9%)	6 (85.7%)		141 (79.2%)	41 (93.2%)		177 (82.3%)	5 (71.4%)	
CNS infiltration	24 (11.5%)	4 (30.8%)	0.065	28 (13.0%)	0 (0.0%)	0.600	27 (15.2%)	1 (2.3%)	0.021^*^	27 (12.6%)	1 (14.3%)	1.000
	Optic disc infiltration			Disc pallor			Venous congestion & tortuosity			Retinal infiltration		
	Absent *N* = 218	Present *N* = 4	*p*	Absent *N* = 218	Present *N* = 4	*p*	Absent *N* = 213	Present *N* = 9	*p*	Absent *N* = 218	Present *N* = 4	*p*
Hb (g/dl)												
Mean ± SD.	8.54 ± 2.04	7.08 ± 2.62	0.157	8.5 ± 2.04	9.25 ± 2.87	0.472	8.56 ± 2.04	7.57 ± 2.33	0.158	8.55 ± 2.05	6.85 ± 2.13	0.102
Blast % in BM												
Mean ± SD.	74.9 ± 22	73.3 ± 25.9	0.886	75.1 ± 21.7	61 ± 34.7	0.205	74.7 ± 22	77.7 ± 21.8	0.693	74.6 ± 22.1	87.8 ± 5.19	0.006*
	Exudative retinal detachment			Ocular motility			Orbital involvement			Macula affection		
	Absent *N* = 218	Present *N* = 4	*p*	Absent *N* = 219	Present *N* = 3	*p*	Absent *N* = 215	Present *N* = 7	*p*	Absent *N* = 217	Present *N* = 5	*p*
Blast % in BM												
Mean ± SD.	74.6 ± 22.1	87.3 ± 3.86	0.001^*^	75 ± 21.9	60 ± 30	0.241	74.9 ± 22	73 ± 23.7	0.825	74.6 ± 22.2	85.2 ± 10.1	0.287

*SD*, standard deviation; *p*, comparing the different categories; * significant

**Fig. 2:**
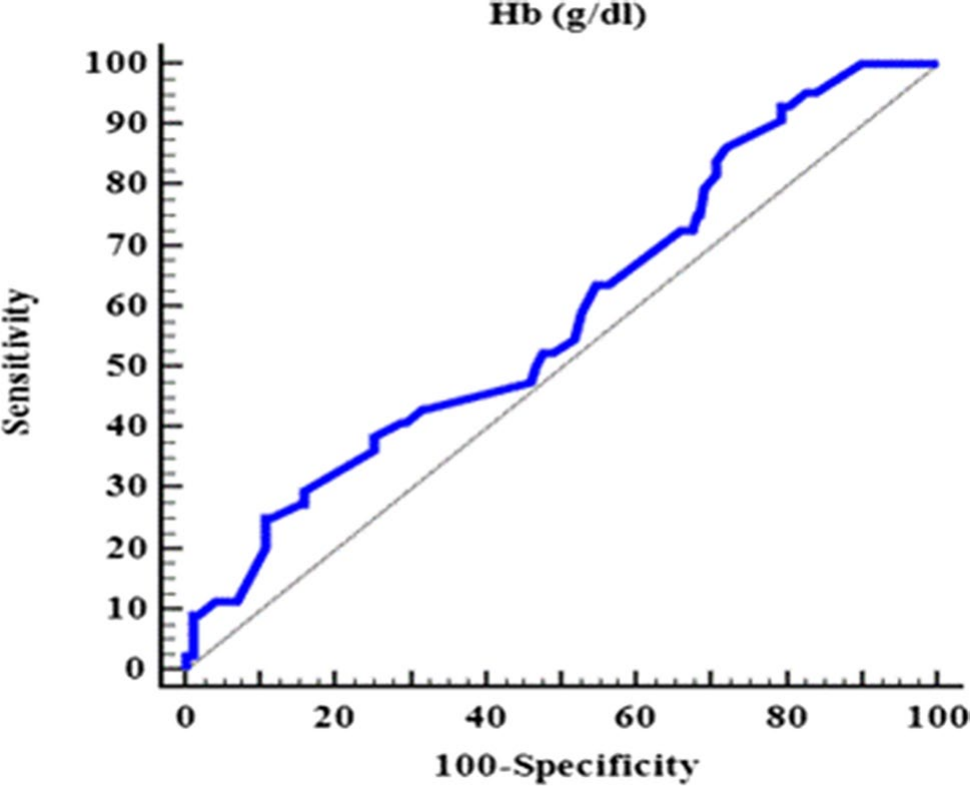
ROC Curve for Hb for discrimination between with and without retinal hemorrhage

It was observed that retinal hemorrhage was significantly associated with acute leukemia patients with lower Hb concentration. In order to predict the likelihood of retinal hemorrhage, a Receiver Operating Characteristic (ROC) curve analysis was conducted with a cut-off value of [≤9.9 g/dL] for Hb concentration. However, the analysis did not yield conclusive results as the Area Under the Curve (AUC) was poor (AUC=0.587, 95% CI 0.495–0.680, *P*=*0.073*) with 86.36% sensitivity and 28.09% specificity. The positive predictive (PPV) and negative predictive values (NPV) were found to be 22.89% and 89.28% respectively, and the overall accuracy was 39.64% (Fig. 2).

The brain radiologic findings were significantly associated with impaired or lost visual acuity, lid swelling, subconjunctival hemorrhage, exudative retinal detachment, and orbital involvement (*P 0.028, 0.019, 0.037, 0.043,* and *0.009*, respectively). Nasal and paranasal sinuses CT findings were significantly associated with lid swelling, impaired ocular motility, and orbital involvement (*P 0.016, 0.035,* and *0.039*, respectively). Otherwise, no significant association was found between brain, nasal, and paranasal sinuses radiological findings and ophthalmic manifestations (Table 4).

**Table 4:** Association between brain, nasal, and paranasal sinuses radiologic findings and ophthalmic manifestations

	Brain radiologic findings			Nasal and paranasal findings		
	Free *N* = 85	Positive *N* = 27	*p*	Free *N* = 66	Positive *N* = 33	*p*
With ophthalmological	44 (51.8%)	15 (55.6%)	0.731	36 (54.5%)	19 (57.6%)	0.775
Visual acuity						
Good	75 (88.2%)	19 (70.4%)	0.028*	56 (84.8%)	25 (75.8%)	0.507
Impaired	9 (10.6%)	5 (18.5%)		8 (12.1%)	6 (18.2%)	
Lost	1 (1.2%)	3 (11.1%)		2 (3.0%)	2 (6.1%)	
Lid ecchymosis	3 (3.5%)	3 (11.1%)	0.150	3 (4.5%)	3 (9.1%)	0.397
Lid ptosis	3 (3.5%)	1 (3.7%)	1.000	1 (1.5%)	3 (9.1%)	0.107
Lid swelling	3 (3.5%)	5 (18.5%)	0.019*	2 (3.0%)	6 (18.2%)	0.016*
Subconjunctival hemorrhage	6 (7.1%)	6 (22.2%)	0.037*	6 (9.1%)	6 (18.2%)	0.207
Conjunctival chemosis	0 (0.0%)	2 (7.4%)	0.056	1 (1.5%)	1 (3.0%)	1.000
Preretinal hemorrhage	1 (1.2%)	1 (3.7%)	0.426	1 (1.5%)	0 (0.0%)	1.000
Retinal hemorrhage	18 (21.2%)	9 (33.3%)	0.198	18 (27.3%)	6 (18.2%)	0.320
Vitreous hemorrhage	2 (2.4%)	3 (11.1%)	0.090	5 (7.6%)	0 (0.0%)	0.166
Roth spots	17 (20.0%)	5 (18.5%)	0.866	14 (21.2%)	6 (18.2%)	0.723
Cotton wool spots	2 (2.4%)	0 (0.0%)	1.000	2 (3.0%)	0 (0.0%)	0.551
Optic disc infiltration	3 (3.5%)	1 (3.7%)	1.000	3 (4.5%)	0 (0.0%)	0.549
Disc pallor	1 (1.2%)	2 (7.4%)	0.144	2 (3.0%)	2 (6.1%)	0.599
Papilledema	3 (3.5%)	1 (3.7%)	1.000	2 (3.0%)	2 (6.1%)	0.599
Venous congestion & tortuosity	3 (3.5%)	1 (3.7%)	1.000	3 (4.5%)	1 (3.0%)	1.000
Retinal infiltration	3 (3.5%)	1 (3.7%)	1.000	2 (3.0%)	0 (0.0%)	0.551
Retinal vein occlusion	1 (1.2%)	0 (0.0%)	1.000	1 (1.5%)	0 (0.0%)	1.000
Exudative retinal detachment	1 (1.2%)	3 (11.1%)	0.043*	4 (6.1%)	0 (0.0%)	0.298
Ocular motility	1 (1.2%)	2 (7.4%)	0.144	0 (0.0%)	3 (9.1%)	0.035*
Orbital involvement	2 (2.4%)	5 (18.5%)	0.009*	2 (3.0%)	5 (15.2%)	0.039*
Macula affection	1 (1.2%)	0 (0.0%)	1.000	2 (3.0%)	0 (0.0%)	0.551
Lagophthalmos	1 (1.2%)	0 (0.0%)	1.000	1 (1.5%)	0 (0.0%)	1.000

p: comparing different categories, *: significant

### Survival analysis

**Fig. 3:**
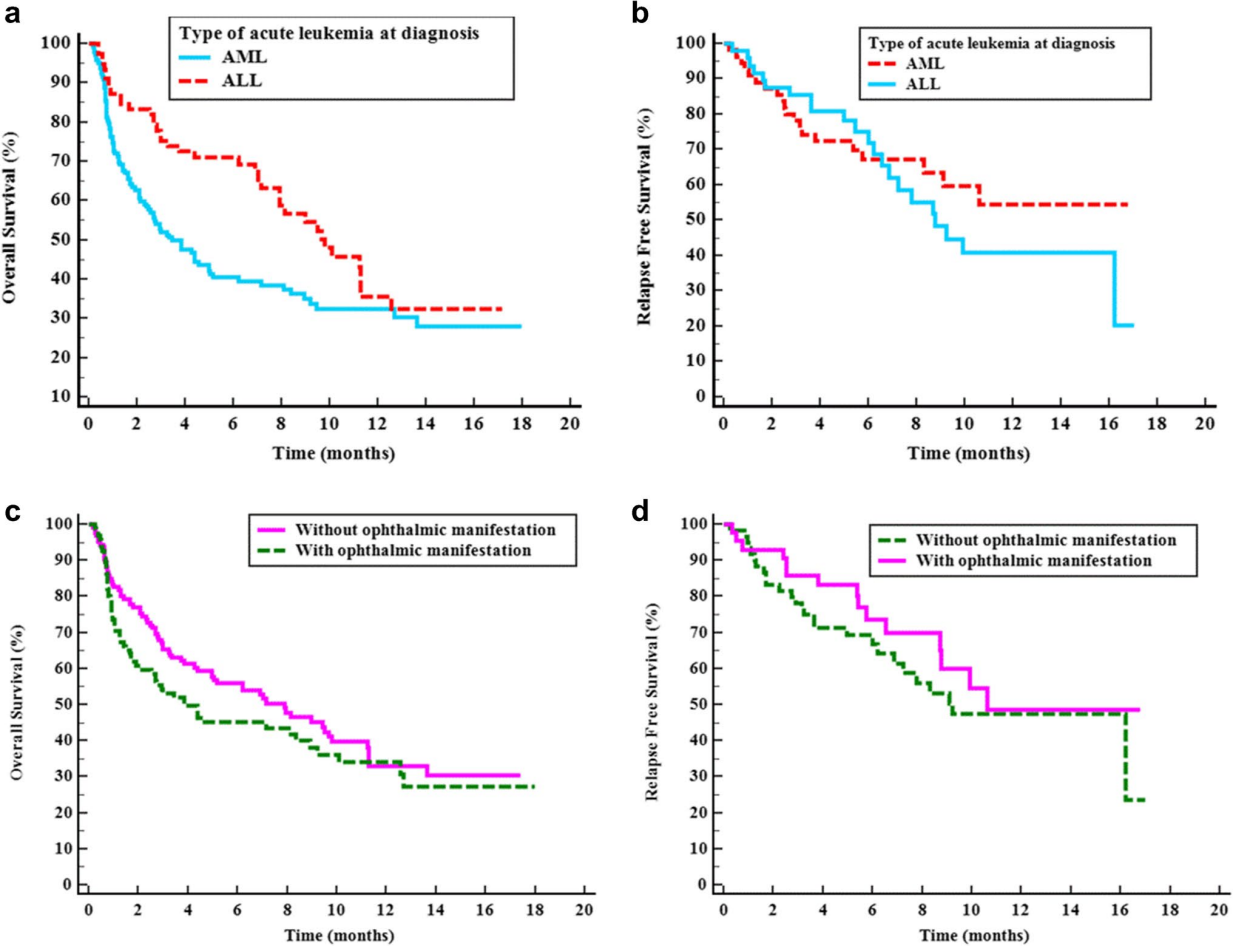
Kaplan Meier studies for Survival analysis for studied acute leukemia patients. **a**) *Overall survival (OS) of newly diagnosed AML and ALL*: 144 AML patients had a mean survival time of 7.38 months, with a standard error of 0.65. The percentage of patients who survived at 6 months, 1 year, and until the end of the study was 40.4%, 32.5%, and 28.1%, respectively. On the other hand, 78 ALL patients had a mean survival time of 9.73 months, with a standard error of 0.78. The percentage of patients who survived at 6 months, 1 year, and until the end of the study was 71.1%, 35.6%, and 32.4%, respectively. There was a significant *P* value of 0.007 between the 2 groups. **b**) *Relapse-free survival (RFS) for AML and ALL patients:* 55 AML patients had a mean relapse-free survival time of 11.17 months, with a standard error of 0.98. The percentage of patients who were relapse-free at 6 months, 1 year, and until the end of the study was 67.3%, 54.4%, and 54.4%, respectively. On the other hand, 48 ALL patients had a mean relapse-free survival time of 10.11 months, with a standard error of 0.98. The percentage of patients who relapse-free at 6 months, 1 year, and until the end of the study was 71.9%, 40.9%, and 20.4%, respectively. There was no significant difference between the 2 groups (*P* value of 0.545). **c**) *OS for acute leukemia patients with and without ophthalmic manifestations:* 126 acute leukemia patients without ophthalmological abnormalities had a mean survival time of 8.26 months, with a standard error of 0.65. The percentage of patients who survived at 6 months, 1 year, and until the end of the study was 55.9%, 33.1%, and 30.5%, respectively. On the other hand, 96 acute leukemia patients with ophthalmological abnormalities had a mean survival time of 7.61 months, with a standard error of 0.8. The percentage of patients who survived at 6 months, 1 year, and until the end of the study was 45.1%, 34%, and 27.2%, respectively. There was no significant difference between the 2 groups (*P* value of 0.192). **d**) *RFS for acute leukemia patients with and without ophthalmic manifestations:* 60 acute leukemia patients without ophthalmological abnormalities had a mean relapse-free survival time of 10.2 months, with a standard error of 0.92. The percentage of patients who were relapse-free at 6 months, 1 year, and until the end of the study was 66.7%, 47.3%, and 23.7%, respectively. On the other hand, 43 acute leukemia patients with ophthalmological abnormalities had a mean relapse-free survival time of 11.26 months, with a standard error of 1.07. The percentage of patients who were relapse-free at 6 months, 1 year, and until the end of the study was 73.7%, 48.5%, and 48.5%, respectively. There was no significant difference between the 2 groups (*P* value of 0.347)

Kaplan Meier test was performed to assess overall survival (OS) and relapse-free survival (RFS) for newly diagnosed AML and ALL patients and the total acute leukemia patients with or without ophthalmological manifestations (Fig. 3). The test showed that ALL patients had significantly better 6-month and 1-year OS than AML patients (71.1 *vs.* 40.4 months and 35.6 *vs.* 32.5 months, respectively (*P=0.007*). However, there was no significant difference found between AML and ALL regarding relapse-free survival (RFS). The study found no difference in OS or RFS between acute leukemia patients with or without ophthalmological manifestations (Fig. 3).

After analyzing the correlation between abnormal ophthalmological findings and early mortality within the first 30 days, we found that only optic disc pallor was significantly associated with early mortality in newly diagnosed acute leukemia patients (*n*=47; *P=0.031*) (Table 5).

**Table 5:** Association between ophthalmic manifestations and early mortality in newly diagnosed acute leukemia patients

	Early mortality		
	No *N* = 175	Yes *N* = 47	*p*
With ophthalmological	71 (40.6%)	25 (53.2%)	0.121
Visual acuity			
Good	161 (92.0%)	42 (89.4%)	0.709
Impaired	11 (6.3%)	4 (8.5%)	
Lost	3 (1.7%)	1 (2.1%)	
Lid ecchymosis	5 (2.9%)	2 (4.3%)	0.641
Lid ptosis	2 (1.1%)	2 (4.3%)	0.198
Lid swelling	6 (3.4%)	3 (6.4%)	0.404
Subconjunctival hemorrhage	8 (4.6%)	5 (10.6%)	0.155
Conjunctival chemosis	1 (0.6%)	1 (2.1%)	0.379
Preretinal hemorrhage	5 (2.9%)	2 (4.3%)	0.641
Retinal hemorrhage	33 (18.9%)	11 (23.4%)	0.487
Vitreous hemorrhage	5 (2.9%)	2 (4.3%)	0.641
Roth spots	29 (16.6%)	9 (19.1%)	0.677
Cotton wool spots	1 (0.6%)	1 (2.1%)	0.379
Optic disc infiltration	3 (1.7%)	1 (2.1%)	1.000
Disc pallor	1 (0.6%)	3 (6.4%)	0.031*
Papilledema	5 (2.9%)	1 (2.1%)	1.000
Venous congestion & tortuosity	7 (4.0%)	2 (4.3%)	1.000
Retinal infiltration	4 (2.3%)	0 (0.0%)	0.581
Retinal vein occlusion	1 (0.6%)	0 (0.0%)	1.000
Exudative retinal detachment	2 (1.1%)	2 (4.3%)	0.198
Ocular motility	2 (1.1%)	1 (2.1%)	0.512
Orbital involvement	6 (3.4%)	1 (2.1%)	1.000
Macula affection	3 (1.7%)	2 (4.3%)	0.286
Lagophthalmos	0 (0.0%)	1 (2.1%)	0.212

p: comparing different categories, *: significant

## Discussion

Acute myeloid leukemia (AML) is an aggressive hematologic malignancy characterized by recurrent cytogenetic and molecular abnormalities leading to abnormal proliferation of myeloid blast cells [13]. Acute lymphoblastic leukemia (ALL) is another heterogeneous malignancy often associated with several chromosomal and molecular abnormalities [14]. Acute leukemia leads to pancytopenia due to bone marrow infiltration, however, it can infiltrate other tissues and lead to extramedullary infiltration in the liver, skin, and CNS, including eyes and orbit [15].

Leukemia can affect any part of the eye. Posterior segment affection particularly retinal hemorrhages are the most commonly reported ocular findings [16]. The estimated prevalence of ophthalmological involvement with acute leukemia is 32% -35.5%.Ocular manifestations can occur in acute leukemia as a result of direct infiltration from leukemic cells or indirect complications induced by cytopenia and hyperviscosity status [17]. These manifestations can be detected in newly diagnosed or even relapsed acute leukemia. Ophthalmological manifestations have been associated with poor prognosis in various studies, while others found no effect on survival [18].

In this study, we detected ophthalmological manifestations in 43.2% of the studied newly diagnosed acute leukemia patients, and bilateral involvement was recorded in 57 (25.7%) patients. This frequency is similar to that reported in previous studies [7, 19, 20], while a higher frequency of eye involvement was observed in other studies ranging from 60% to 90% [6, 21, 22].

The incidence of ophthalmological manifestations was significantly higher in patients with AML than in those with ALL patients (72/144 (50%) *vs.* 24/78 (30.7%), respectively). Additionally, AML patients had a higher frequency of left eye and bilateral affection (*P 0.028*), retinal hemorrhage (*P 0.003*), and Roth spots (*P 0.046*) compared to ALL patients. Previous studies have also demonstrated that ophthalmic findings were more common in AML than ALL [7] and retinal hemorrhages have been also reported more commonly in Tunisian AML patients [22].

Consistent with our findings Jihene Sayadi et.al [22], .have illustrated that retinal hemorrhages were significantly associated with lower Hb concentrations (*P 0.038*) and thrombocytopenia (*P0.035*), however, in our study we did not find a significant association between retinal hemorrhage and thrombocytopenia or leukocytosis (*P 0.283*and *0.38*, respectively) in contrast to conclusions delivered by Malaysian study showing that leukocytosis and thrombocytopenia were significantly associated with retinal hemorrhages in AML patients [23].

In our cohort, ERD was detected in 4 AML patients and was significantly associated with higher BM blast percentage [87.5% (range, 83 – 91), *P 0.001*]. Retinal detachment has been reported as a presenting finding of AML in adults and children. Serious hemorrhagic retinal detachment has been described in a patient with chronic myeloid leukemia (CML) presenting with severe visual loss secondary to hyperleukocytosis that lead to retinal circulatory stasis and ischemia [24]. The suggested theory is that leukemic choroidal infiltration causes a decrease in blood flow to the choriocapillaris, leading to ischemia of the retinal pigment epithelium (RPE) and subsequently disrupting the inter-cellular tight junctions. RPE fails to pump fluid and ERD occurs [25].

Consistent with our findings, the Tunisian study did not detect a correlation between blood count parameters and cotton wool or Roth spots [22]. Roth spots [26] and cotton wool spots [27] are considered as leukemic retinopathy, and they are usually asymptomatic.

Orbital involvement in acute leukemia has a variable presentation including eyelid swelling, ptosis or more commonly proptosis. Some studies did not find a prognostic impact of orbital involvement in acute leukemia [18] which is consistent with our results, while others have illustrated a poor prognostic association in AML [28, 29].

Optic disc edema can result from direct infiltration or secondary to leukemic CNS involvement. Patients with suspected leukemic CNS infiltration e.g., having neurological and/or ophthalmological signs and symptoms are assigned for lumbar puncture and CSF evaluation by conventional or FCM analysis to detect leukemic infiltration after exclusion of hemorrhage or space-occupying lesions by radiology. Optic disc infiltration was documented in 4 patients without proven CSF infiltration and was not detected in patients with CSF infiltration, no significant association was found between CNS infiltration with optic disc infiltration or papilledema (Table 6), while retinal hemorrhage was significantly associated with CNS infiltration (Table 3).

**Table 6:** Association between CNS infiltration with leukemic eye infiltration and papilledema

	CNS infiltration		*p*
	No *N* = 194	Yes *N* = 28	
Optic disc infiltration	4 (2.1%)	0 (0.0%)	1.000
Retinal infiltration	3 (1.5%)	1 (3.6%)	0.419
Papilledema	4 (2.1%)	2 (7.1%)	0.167

p: comparing different categories, *: significant

The ophthalmological manifestations detected in our patients can be summarized into 3 categories as shown in (Table 7). Treatment details for patients in category I “Direct infiltration of the anterior segment, vitreous, choroid, and retina mimicking uveitis, choroiditis and retinitis” are illustrated in (Table 8), 65.4% of these patients were AML and received 3+7 protocol at induction therapy (*P 0.004*). There was no significant difference as regard response to induction chemotherapy between patients with or without the manifestations grouped in category I.

**Table 7:** Classification of ophthalmological findings detected in studied acute leukemia patients

*Category I.* Direct infiltration of the anterior segment, vitreous, choroid, and retina mimicking uveitis, choroiditis, and retinitis		81 (36.5%)
*Category II.* Infiltration of the optic nerve presenting with or without another cranial nerve involvement clinically mimicking palsies and swollen discs (*n*=15)	A. Infiltration of the optic nerve without another cranial nerve involvement	14 (6.3%)
	B. Infiltration of the optic nerve with another cranial nerve involvement (Cranial nerve VII)	1 (0.5%)
*Category III*. Infiltration of the orbit mimicking orbital inflammatory diseases		11 (5%)

**Table 8:** Association of direct infiltration of the anterior segment, vitreous, choroid, and retina mimicking uveitis, choroiditis, and retinitis with treatment

		Direct infiltration of the anterior segment, vitreous, choroid, and retina mimicking uveitis, choroiditis, and retinitis				*p*
		Absent		Present		
		*N*	%	*N*	%	
Type of induction treatment	BSC	14	9.9%	6	7.4%	0.004*
	Standard intensity for AML	52	36.9%	53	65.4%	
	Low intensity for AML	20	14.2%	6	7.4%	
	Standard intensity for ALL	24	17.0%	8	9.9%	
	Pediatric inspired for ALL	27	19.1%	7	8.6%	
	Low intensity for ALL	4	2.8%	1	1.2%	
TKI therapy	No	126	89.4%	75	92.6%	0.428
	yes	15	10.6%	6	7.4%	
Response to induction treatment	CR	63	44.7%	35	43.2%	0.341
	PR	6	4.3%	1	1.2%	
	Refractory	24	17.0%	15	18.5%	
	Induction death	18	12.8%	17	21.0%	
	Not applicable	30	21.3%	13	16.0%	
Type of salvage for R/R	No salvage	110	78.0%	65	80.2%	0.550
	HAM or FLAG	27	19.1%	12	14.8%	
	Low intensity therapy	4	2.8%	4	4.9%	
Response to salvage	CR	14	9.9%	4	4.9%	0.419
	PR	14	9.9%	8	9.9%	
	Not applicable	113	80.1%	69	85.2%	
IT chemotherapy for CNS infiltration	no	122	86.5%	72	88.9%	0.610
	yes	19	13.5%	9	11.1%	
Response to IT chemotherapy	NR	4	21.1%	1	11.1%	0.346
	CR	12	63.2%	4	44.4%	
	not applicable	3	15.8%	4	44.4%	

p: comparing different categories, *: significant. *BSC* best supportive care, *TKI* tyrosine kinase inhibitor, *CR* complete response, *PR* partial response, *R/R* relapsed/refractory, *IT* intrathecal, *CNS* central nervous system, *NR* no response

In terms of ophthalmological findings in acute leukemia the treatment was entirely depending on systemic antileukemic measures together with supportive treatment like blood transfusion products especially in hemorrhagic manifestations or broad-spectrum antibiotics if underlying infection was suspected, ophthalmological treatment was only in the form of supportive measures like local anti-inflammatory or antibiotic medications. Surgical interference was performed in one case with ERD.

A previous retrospective study conducted on patients with intraocular leukemia of different etiologies recommended repeated intravitreal methotrexate injections as an adjuvant therapy in combination with IT chemotherapy in leukemic patients with medullary remission, their results showed improvement of the inflammatory reactions, resolution of swollen disc, resolution of retinal and disc tumor cell infiltrates, however, there was no improvement in retinal hemorrhages [30].

The study herein did not illustrate a significant difference between acute leukemia patients with and without ophthalmological findings as regard the OS (7.6 *vs.* 8.6 months, respectively (*P 0.192*)) and RFS analysis (11.26 *vs.* 10.26 months, respectively (*P 0.347*)) in contrary to what was concluded by Mirashi and colleagues; who found that ocular findings were associated with increased mortality, especially on the first day after diagnosis [31].

In our study, optic disc pallor was the only parameter which was significantly associated with early mortality (first 30-day mortality after diagnosis) (*P 0.031*). Another study has also demonstrated no significant differences in survival analysis between acute leukemia patients with or without ocular signs (*P 0.778*) [22]. Previous studies have concluded that ophthalmological manifestations in patients with leukemias were associated with poor prognosis and dismal survival [32–34].

Our study has some limitations, primarily due to the relatively small sample size, especially for ALL cases. The main objective of our study was to determine the prevalence of ocular manifestations at the time of diagnosis of acute leukemia and to examine its correlation with disease characteristics and course. Moreover, the lack of follow-up data is a significant limitation for now. Therefore, we plan to expand our research by conducting follow-up studies on ophthalmological manifestations after chemotherapy and at the end of patients’ treatment to evaluate the long-term effects of these findings on patients’ prognosis and quality of life.

## Conclusion

Ophthalmological manifestations of acute leukemia are heterogeneous; they can be detected at initial presentations or relapse. Some manifestations are asymptomatic, others may have an impact on visual acuity thus altering the patient’s quality of life, or even the disease course, especially if those associated with CNS infiltration as this could require modifications in the treatment plan for incorporation of high-dose chemotherapies that cross blood-brain barrier and assessment for stem cell transplantation. Cooperation between ophthalmologists and haemato-oncologists is crucial for recognizing ocular involvement and disease management. We need further evaluation of a larger cohort of acute leukemia patients especially for survival analysis to set the record for the prognostic value of ocular manifestations in such neoplasms.

### Supplementary information

ESM 1 (DOCX 64 kb)

## Data Availability

No datasets were generated or analysed during the current study.

## Abbreviations

AL: Acute Leukemia
ALL: Acute Lymphoblastic Leukemia
AML: Acute Myeloid Leukemia
AUC: Area Under The Curve
BSC: Best Supportive Care
BM: Bone Marrow
CML: Chronic myeloid leukemia
CNS: Central Nervous System
CSF: Cerebrospinal Fluid
CBC: Complete Blood Counts
CR: Complete Remission
ELN: EuropeanLeukemianet
ERD: Exudative retinal detachment
FCM: Flowcytometry
Hb: Hemoglobin
HMAs: Hypomethylating Agents
ICT: Intensive Chemotherapy Treatment
IT: Intrathecal
LDAC: Low-Dose Cytarabine
MOC: Mansoura Ophthalmic Center
NPV: Negative Predictive Values
OCMU: Oncology Center Mansoura University
OS: Overall Survival
PR: Partial Remission
PPV: Positive Predictive
R/R: Relapsed/Refractory
RFS: Relapse-Free Survival
ROC: Receiver Operating Characteristic
3D DRI OCT: Three-Dimensional Deep Range Imaging Optical Coherence Tomography
TKI: Tyrosine Kinase Inhibitors
UDVA: Uncorrected Distance Visual Acuity

## References

[1] Tebbi CK (2021) Etiology of acute leukemia: A review. Cancers 13(9):2256 34066700 10.3390/cancers13092256 PMC8125807

[2] Juliusson G, Antunovic P, Derolf Å, Lehmann S, Möllgård L, Stockelberg D et al (2009) Age and acute myeloid leukemia: real world data on decision to treat and outcomes from the Swedish Acute Leukemia Registry. Blood 113(18):4179–4187 19008455 10.1182/blood-2008-07-172007

[3] Howlader N, Noone A, Krapcho M. National Cancer Institute. SEER Cancer Statistics Review (CSR) 1975-2014. 2021.

[4] Swerdlow SH, Campo E, Harris NL, Jaffe ES, Pileri SA, Stein H, et al. WHO classification of tumours of haematopoietic and lymphoid tissues: International agency for research on cancer Lyon, France; 2008.

[5] Vardiman J, Hyjek E. World health organization classification, evaluation, and genetics of the myeloproliferative neoplasm variants. Hematology 2010, the American Society of Hematology Education Program Book. 2011;2011(1):250-6. 10.1182/asheducation-2011.1.250 22160042

[6] Talcott KE, Garg RJ, Garg SJ (2016) Ophthalmic manifestations of leukemia. Curr Opin Ophthalmol 27(6):545–551 27585213 10.1097/ICU.0000000000000309

[7] Hafeez MU, Ali MH, Najib N, Ayub MH, Shafi K, Munir M et al (2019) Ophthalmic manifestations of acute leukemia. Cureus 11(1) 10.7759/cureus.3837 PMC6411336 30891378

[8] Soman S, Kasturi N, Srinivasan R, Vinod K (2018) Ocular manifestations in leukemias and their correlation with hematologic parameters at a tertiary care setting in South India. Ophthalmol Retina 2(1):17–23 31047297 10.1016/j.oret.2017.05.009

[9] Bukhari ZM, Alzahrani A, Alqarni MS, Alajmi RS, Alzahrani A, Almarzouki H et al (2021) Ophthalmic manifestations in acute leukemia patients and their relation with hematological parameters in a tertiary care center. Cureus 13(11) 10.7759/cureus.19384 PMC8655321 34925986

[10] Dhasmana R, Prakash A, Gupta N, Verma S (2016) Ocular manifestations in leukemia and myeloproliferative disorders and their association with hematological parameters. Ann Afr Med 15(3):97 27549412 10.4103/1596-3519.188887 PMC5402809

[11] Daniel WW, Cross CL (2018) Biostatistics: a foundation for analysis in the health sciences. Wiley

[12] Döhner H, Estey E, Grimwade D, Amadori S, Appelbaum FR, Büchner T et al (2017) Diagnosis and management of AML in adults: 2017 ELN recommendations from an international expert panel. Blood 129(4):424–447 27895058 10.1182/blood-2016-08-733196 PMC5291965

[13] Lachowiez CA, Long N, Saultz J, Gandhi A, Newell LF, Hayes-Lattin B et al (2023) Comparison and validation of the 2022 European LeukemiaNet guidelines in acute myeloid leukemia. Blood Adv 7(9):1899–1909 36441905 10.1182/bloodadvances.2022009010 PMC10172873

[14] Frikha R, El Aoud A, Kamoun H (2023) ALL-022 Assessment of Molecular Response to Tyrosine Kinase Inhibitors in Tunisian Patients With Ph+ Acute Lymphoblastic Leukemia. Clin Lymphoma Myeloma Leuk 23:S237 10.1016/S2152-2650(23)00928-X

[15] Lowenberg B, Downing JR, Burnett A (1999) Acute myeloid leukemia. N Engl J Med 341(14):1051–1062 10502596 10.1056/NEJM199909303411407

[16] Sharma H, Majumder PD, Rao C, Biswas J (2016) A case of acute myeloid leukemia masquerading as unilateral exudative detachment. Am J Ophthalmol Case Rep 4:47–49 29503924 10.1016/j.ajoc.2016.08.004 PMC5757459

[17] Binkley EM, Schachat AP (2022) Leukemias. Albert and Jakobiec's Principles and Practice of Ophthalmology. Springer, pp 7775–7782

[18] El Salloukh NA, Hage DG, Bashshur AZ, Kheir WJ (2022) Early Ophthalmological Manifestations of Acute Myeloid Leukemia: Current Perspectives. Clin Ophthalmol:2119–2127 10.2147/OPTH.S342720 PMC9255417 35800672

[19] Khadka D, Sharma A, Shrestha JK, Shrestha GS, Shrestha PN, Pant SR et al (2014) Ocular manifestations of childhood acute leukemia in a tertiiary level eye centre of Kathmandu, Nepal. Nepal J Ophthalmol 6(2):197–204 25680250 10.3126/nepjoph.v6i2.11678

[20] Koshy J, John MJ, Thomas S, Kaur G, Batra N, Xavier WJ (2015) Ophthalmic manifestations of acute and chronic leukemias presenting to a tertiary care center in India. Indian J Ophthalmol 63(8):659 26576524 10.4103/0301-4738.169789 PMC4687193

[21] Gawai D, Jhavar S, Patil S (2016) Orbital and ocular manifestations of acute and chronic leukemia. Int J Health Sci Res 6(9):61–64

[22] Sayadi J, Gouider D, Allouche Y, Choura R, Cherni I, Sayadi M et al (2022) Ophthalmic Manifestations of Newly Diagnosed Acute Leukemia Patients in a Tunisian Cohort. Clin Ophthalmol:3425–3435 10.2147/OPTH.S365648 PMC9560867 36249442

[23] Reddy S, Jackson N (2004) Retinopathy in acute leukaemia at initial diagnosis: correlation of fundus lesions and haematological parameters. Acta Ophthalmol Scand 82(1):81–85 14738490 10.1046/j.1600-0420.2003.00197.x

[24] Chen B, Yan X, Zhang X, Yang H (2018) Leukostasis retinopathy: An uncommon visual threatening complication of chronic myeloid leukemia with severe hyperleukocytosis – A case report and review of the literature. Indian J Ophthalmol 66(12):1871–1874 30451209 10.4103/ijo.IJO_627_18 PMC6256890

[25] Naseripour M, Abdolalizadeh P, Abdi F, Mehrvar A, Tashvighi M (2019) Serous retinal detachment as an initial presentation of childhood acute myeloid leukemia. Can J Ophthalmol 54(4):e170–e1e3 31358158 10.1016/j.jcjo.2018.10.008

[26] Chandra A, Chakraborty U, Ganai S, Ray AK (2020) Roth spots in acute myeloid leukaemia. BMJ Case Rep 13(9) 10.1136/bcr-2020-238133 PMC7470486 32878844

[27] Shyam S (2023) Study of ocular manifestations in leukemia: a cross-sectional study. Int J Acad Med Pharm 5(4):90–94

[28] Yaghouti F, Nouri M, Mannor GE (1999) Ocular adnexal granulocytic sarcoma as the first sign of acute myelogenous leukemia relapse. Am J Ophthalmol 127(3):361–363 10088758 10.1016/S0002-9394(98)00363-8

[29] Adenis T, Labrousse F, Sauvage J, Robert P (2006) Granulocytic sarcoma in a 90-year-old patient. J Fr Ophtalmol 29(8):961–964 17075518 10.1016/S0181-5512(06)70128-0

[30] Vishnevskia-Dai V, Sella King S, Lekach R, Fabian ID, Zloto O (2020) Ocular Manifestations of Leukemia and Results of Treatment with Intravitreal Methotrexate. Sci Rep 10(1):1994 32029770 10.1038/s41598-020-58654-8 PMC7005017

[31] Mirshahi R, Ghassemi F, Koochakzadeh L, Faranoush M, Ghomi Z, Mehrvar A et al (2022) Ocular manifestations of newly diagnosed acute leukemia patients. J Curr Ophthalmol 34(1):100 35620369 10.4103/joco.joco_10_21 PMC9128435

[32] Russo V, Scott I, Querques G, Stella A, Barone A, Delle NN (2008) Orbital and ocular manifestations of acute childhood leukemia: clinical and statistical analysis of 180 patients. Eur J Ophthalmol 18(4):619–623 18609485 10.1177/112067210801800420

[33] Němčanská S, Stepanov A, Němčanský J (2018) Ophthalmic manifestations of acute leukaemias. Cesk Slov Oftalmol 74(3):98–101 30650972 10.31348/2018/1/3-3-2018

[34] Chefchaouni C, Belmekki M, Hajji Z, Tahiri H, Amrani R, El Bakkali M et al (2002) Ophthalmic manifestations of acute leukemia. J Fr Ophtalmol 25(1):62–66 11965121

