# Supplementary material for: Highlights of ophthalmological manifestations in newly diagnosed acute leukemia: a correlation with hematological parameters
Source: Ann Hematol. 2024 Jul 10;103(9):3519–33. doi: 10.1007/s00277-024-05861-2 (PMC11358343; doi:10.1007/s00277-024-05861-2)
Supplement: Supplementary file 1 — (DOCX 64 kb) [file 277_2024_5861_MOESM1_ESM.docx]

***Supplementary Table 1. Brain Radiology, Nasal and Paranasal sinuses Radiology.***

| **Radiology** | | **N** | **%** |
| --- | --- | --- | --- |
| **Brain** | Free | 85 | 38.3% |
|  | Infarction | 2 | 0.9% |
|  | Age related brain changes | 3 | 1.4% |
|  | Ischemic changes | 2 | 0.9% |
|  | Not evaluated | 110 | 49.5% |
|  | White matter atherosclerosis | 1 | 0.5% |
|  | Orbital &/or ophthalmological changes | 2 | 0.9% |
|  | SOL or brain infiltration | 5 | 2.3% |
|  | Brain or periorbital abscess | 2 | 0.9% |
|  | Brain hemorrhage | 4 | 1.8% |
|  | Brain involutional changes | 1 | 0.5% |
|  | Thrombosis | 1 | 0.5% |
|  | Age related and ischemic brain changes | 2 | 0.9% |
|  | SOL or brain infiltration and brain hemorrhage | 1 | 0.5% |
|  | Orbital &/or ophthalmological changes and brain or periorbital abscess | 1 | 0.5% |
| **Nasal and Paranasal sinuses** | Free | 66 | 29.7% |
|  | Sinusitis | 19 | 8.6% |
|  | Invasive fungal sinusitis | 1 | 0.5% |
|  | Preseptal cellulitis | 0 | 0.0% |
|  | Deviated nasal septum | 0 | 0.0% |
|  | Nasal & paranasal soft tissue infiltration | 7 | 3.2% |
|  | Not evaluated | 123 | 55.4% |
|  | Invasive fungal sinusitis and nasal & paranasal soft tissue infiltration | 1 | 0.5% |
|  | Sinusitis and nasal & paranasal soft tissue infiltration | 1 | 0.5% |
|  | Sinusitis and deviated nasal septum | 2 | 0.9% |
|  | Sinusitis and preseptal cellulitis and nasal & paranasal soft tissue infiltration | 1 | 0.5% |
|  | Sinusitis and deviated nasal septum and nasal & paranasal soft tissue infiltration | 1 | 0.5% |

***Supplementary Table 2. Association between visual acuity, lid ecchymosis, ptosis, and swelling with different parameters***

|  | **Visual acuity** | | | **Lid ecchymosis** | | | **Lid ptosis** | | | **Lid swelling** | | |
| --- | --- | --- | --- | --- | --- | --- | --- | --- | --- | --- | --- | --- |
|  | **Good N = 203** | **impaired+ lost N = 19** | **p** | **Absent N = 215** | **Present N = 7** | **p** | **Absent N = 218** | **Present N = 4** | **p** | **Absent N = 213** | **Present N = 9** | **p** |
| **Hb (g/dl)** |  |  |  |  |  |  |  |  |  |  |  |  |
| Mean ± SD. | 8.48 ± 2.06 | 8.87 ± 1.98 | 0.428 | 8.5 ± 2.07 | 8.93 ± 1.59 | 0.590 | 8.54 ± 2.07 | 7.38 ± 0.75 | 0.263 | 8.5 ± 2.07 | 8.93 ± 1.81 | 0.535 |
| Median (Min. – Max.) | 8.5(3 – 15) | 8.5(5 – 13) |  | 8.5(3 – 15) | 9.5(7 – 11) |  | 8.5(3 – 15) | 7(7 – 8.5) |  | 8.5(3 – 15) | 8.7(7 – 11.7) |  |
| **TLC (k/uL)** |  |  |  |  |  |  |  |  |  |  |  |  |
| Mean ± SE. | 51.3 ± 5.48 | 95.4 ± 27.1 | 0.185 | 55.99 ± 5.71 | 25.9 ± 14.1 | 0.460 | 54.7 ± 5.58 | 71.7 ± 57 | 0.669 | 56.4 ± 5.76 | 23.23 ± 11 | 0.347 |
| Median (Min. – Max.) | 18(0.3 – 670) | 39(0.4 – 449) |  | 19(0.3 – 670) | 9.5(2.2 – 104) |  | 19(0.3 – 670) | 23(0.4 – 240) |  | 19(0.3 – 670) | 10(0.4 – 104) |  |
| **Plt count (k/uL)** |  |  |  |  |  |  |  |  |  |  |  |  |
| Mean ± SE. | 57.5 ± 4.64 | 61.1 ± 17.1 | 0.658 | 56.9 ± 4.55 | 82.7 ± 25.6 | 0.306 | 56.7 ± 4.39 | 115.5 ± 70.6 | 0.390 | 57.5 ± 4.59 | 64.6 ± 21.4 | 0.705 |
| Median (Min. – Max.) | 32(2 – 512) | 39(5.8 – 322) |  | 32(2 – 512) | 88(13 – 179) |  | 32(2 – 512) | 64(13 – 322) |  | 32(2 – 512) | 33(13 – 179) |  |
| **Blast % in PB** |  |  |  |  |  |  |  |  |  |  |  |  |
| Mean ± SE. | 60.1 ± 1.79 | 64.8 ± 6.58 | 0.306 | 60.6 ± 1.74 | 58.9 ± 13.2 | 0.850 | 60.5 ± 1.75 | 64 ± 15.3 | 0.774 | 60.1 ± 1.75 | 70 ± 10.5 | 0.202 |
| Median (Min. – Max.) | 60(5 – 98) | 73(6 – 95) |  | 60(5 – 98) | 84(6 – 89) |  | 60(5 – 98) | 65.5(30 – 95) |  | 60(5 – 98) | 86(6 – 95) |  |
| **Blast % in BM** |  |  |  |  |  |  |  |  |  |  |  |  |
| Mean ± SD. | 75 ± 21.7 | 72.8 ± 25.9 | 0.683 | 75.1 ± 21.9 | 67 ± 26.9 | 0.340 | 75.2 ± 21.7 | 52.5 ± 28.7 | 0.040^*^ | 75 ± 21.9 | 71.3 ± 25.3 | 0.628 |
| Median (Min. – Max.) | 85(6 – 98) | 90(24 – 95) |  | 85(6 – 98) | 77(30 – 90) |  | 85(6 – 98) | 45(30 – 90) |  | 85(6 – 98) | 90(30 – 90) |  |
| **Cytogenetics** | 59 (29.1%) | 5 (26.3%) | 0.800 | 61 (28.4%) | 3 (42.9%) | 0.414 | 63 (28.9%) | 1 (25.0%) | 1.000 | 60 (28.2%) | 4 (44.4%) | 0.284 |
| **Molecular abnormality** | 18 (8.9%) | 1 (5.3%) | 1.000 | 18 (8.4%) | 1 (14.3%) | 0.470 | 18 (8.3%) | 1 (25.0%) | 0.303 | 17 (8.0%) | 2 (22.2%) | 0.174 |
| **BCR/ABL1** | 17 (8.4%) | 4 (21.1%) | 0.089 | 21 (9.8%) | 0 (0.0%) | 1.000 | 20 (9.2%) | 1 (25.0%) | 0.330 | 21 (9.9%) | 0 (0.0%) | 1.000 |
| **Risk Stratification** |  |  |  |  |  |  |  |  |  |  |  |  |
| Favorable | 66 (32.5%) | 5 (26.3%) | 0.438 | 68 (31.6%) | 3 (42.9%) | 0.889 | 70 (32.1%) | 1 (25.0%) | 1.000 | 70 (32.9%) | 1 (11.1%) | 0.400 |
| Intermediate | 80 (39.4%) | 6 (31.6%) |  | 84 (39.1%) | 2 (28.6%) |  | 84 (38.5%) | 2 (50.0%) |  | 81 (38.0%) | 5 (55.6%) |  |
| Poor | 57 (28.1%) | 8 (42.1%) |  | 63 (29.3%) | 2 (28.6%) |  | 64 (29.4%) | 1 (25.0%) |  | 62 (29.1%) | 3 (33.3%) |  |
| **Response to induction** |  |  |  |  |  |  |  |  |  |  |  |  |
| CR | 91 (44.8%) | 7 (36.8%) | 0.623 | 95 (44.2%) | 3 (42.9%) | 0.527 | 97 (44.5%) | 1 (25.0%) | 0.360 | 96 (45.1%) | 2 (22.2%) | 0.460 |
| PR | 6 (3.0%) | 1 (5.3%) |  | 7 (3.3%) | 0 (0.0%) |  | 7 (3.2%) | 0 (0.0%) |  | 7 (3.3%) | 0 (0.0%) |  |
| Refractory | 34 (16.7%) | 5 (26.3%) |  | 37 (17.2%) | 2 (28.6%) |  | 39 (17.9%) | 0 (0.0%) |  | 36 (16.9%) | 3 (33.3%) |  |
| Induction death | 33 (16.3%) | 2 (10.5%) |  | 33 (15.3%) | 2 (28.6%) |  | 34 (15.6%) | 1 (25.0%) |  | 33 (15.5%) | 2 (22.2%) |  |
| Not applicable | 39 (19.2%) | 4 (21.1%) |  | 43 (20.0%) | 0 (0.0%) |  | 41 (18.8%) | 2 (50.0%) |  | 41 (19.2%) | 2 (22.2%) |  |
| **Relapse for CR** | 23/91(25.3%) | 3/7 (42.9%) | 0.378 | 26/95(27.4%) | 0/3(0.0%) | 0.563 | 26/97(26.8%) | 0/1(0.0%) | 1.000 | 26/96(27.1%) | 0/2(0.0%) | 1.000 |
| **Response to salvage** |  |  |  |  |  |  |  |  |  |  |  |  |
| CR | 16 (7.9%) | 2 (10.5%) | 0.510 | 18 (8.4%) | 0 (0.0%) | 0.756 | 18 (8.3%) | 0 (0.0%) | 1.000 | 18 (8.5%) | 0 (0.0%) | 0.359 |
| PR | 19 (9.4%) | 3 (15.8%) |  | 21 (9.8%) | 1 (14.3%) |  | 22 (10.1%) | 0 (0.0%) |  | 20 (9.4%) | 2 (22.2%) |  |
| Not applicable | 168 (82.8%) | 14 (73.7%) |  | 176 (81.9%) | 6 (85.7%) |  | 178 (81.7%) | 4 (100.0%) |  | 175 (82.2%) | 7 (77.8%) |  |
| **CNS infiltration** | 24 (11.8%) | 4 (21.1%) | 0.273 | 25 (11.6%) | 3 (42.9%) | 0.045^*^ | 27 (12.4%) | 1 (25.0%) | 0.419 | 25 (11.7%) | 3 (33.3%) | 0.090 |

SD.: Standard deviation, SE.: Standard error, Min.: Minimum, Max.: Maximum, t: Student t-test, U: Mann–Whitney, X^2^: Chi-Square, FE: Fisher Exact, MC: Monte Carlo, P: Comparing the different categories, *: Significant.

***Supplementary Table 3. Association between subconjunctival, preretinal, retinal, and vitreous hemorrhage with different parameters***

|  | **Subconjunctival hemorrhage** | | | **Preretinal hemorrhage** | | | **Retinal hemorrhage** | | | **Vitreous hemorrhage** | | |
| --- | --- | --- | --- | --- | --- | --- | --- | --- | --- | --- | --- | --- |
|  | **Absent N = 209** | **Present N = 13** | **p** | **Absent N = 215** | **Present N = 7** | **p** | **Absent N = 178** | **Present N = 44** | **p** | **Absent N = 215** | **Present N = 7** | **p** |
| **Hb (g/dl)** |  |  |  |  |  |  |  |  |  |  |  |  |
| Mean ± SD. | 8.5 ± 2.06 | 8.78 ± 2.1 | 0.628 | 8.5 ± 2.08 | 9.07 ± 1.09 | 0.468 | 8.67 ± 2.05 | 7.88 ± 1.96 | 0.021^*^ | 8.54 ± 2.07 | 7.86 ± 1.36 | 0.390 |
| Median (Min. – Max.) | 8.5(3 – 15) | 8(5 – 11.7) |  | 8.4(3 – 15) | 9(7.5 – 10.4) |  | 8.5(3.5 – 15) | 8.25(3 – 11) |  | 8.5(3 – 15) | 8.3(5 – 9) |  |
| **TLC (k/uL)** |  |  |  |  |  |  |  |  |  |  |  |  |
| Mean ± SE. | 55.7 ± 5.84 | 44.4 ± 14.5 | 0.843 | 55.3 ± 5.7 | 45.9 ± 22.2 | 0.704 | 58.4 ± 6.59 | 41.6 ± 8.48 | 0.380 | 55 ± 5.69 | 56.9 ± 25.1 | 0.389 |
| Median (Min. – Max.) | 19(0.3 – 670) | 18(0.4 – 146) |  | 18(0.3 – 670) | 23(2.3 – 171) |  | 19(0.3 – 670) | 15(0.4 – 237) |  | 19(0.3 – 670) | 38(6 – 194) |  |
| **Plt count (k/uL)** |  |  |  |  |  |  |  |  |  |  |  |  |
| Mean ± SE. | 58 ± 4.65 | 54 ± 16.6 | 0.487 | 58.8 ± 4.6 | 26.5 ± 4.97 | 0.220 | 60.8 ± 5.19 | 45.6 ± 8.23 | 0.283 | 58.8 ± 4.6 | 24.7 ± 4.58 | 0.131 |
| Median (Min. – Max.) | 32(2 – 512) | 24(10 – 179) |  | 33(2 – 512) | 25(10 – 48) |  | 33.5(2 – 512) | 29(5.8 – 320) |  | 33(2 – 512) | 21(11 – 48.6) |  |
| **Blast % in PB** |  |  |  |  |  |  |  |  |  |  |  |  |
| Mean ± SE. | 59.88 ± 1.79 | 71.08 ± 5.89 | 0.181 | 60.50 ± 1.77 | 61.43 ± 8.35 | 0.933 | 60.37 ± 1.99 | 61.20 ± 3.45 | 0.992 | 60.49 ± 1.77 | 61.71 ± 6.94 | 0.993 |
| Median (Min. – Max.) | 60(5 – 98) | 80(32 – 95) |  | 60(5 – 98) | 58(31 – 90) |  | 62.50(5 – 98) | 60(20 – 95) |  | 60(5 – 98) | 60(35 – 90) |  |
| **Blast % in BM** |  |  |  |  |  |  |  |  |  |  |  |  |
| Mean ± SD. | 74.7 ± 22.1 | 76.9 ± 21.8 | 0.733 | 74.8 ± 22.1 | 76 ± 20 | 0.886 | 75 ± 22 | 74.3 ± 22.1 | 0.854 | 74.7 ± 22 | 78.3 ± 23.7 | 0.673 |
| Median (Min. – Max.) | 85(6 – 98) | 90(30 – 92) |  | 85(6 – 98) | 85(36 – 95) |  | 85(6 – 98) | 83(20 – 97) |  | 85(6 – 98) | 85(27 – 97) |  |
| **Cytogenetics** | 57 (27.3%) | 7 (53.8%) | 0.056 | 63 (29.3%) | 1 (14.3%) | 0.676 | 55 (30.9%) | 9 (20.5%) | 0.171 | 61 (28.4%) | 3 (42.9%) | 0.414 |
| **Molecular abnormality** | 16 (7.7%) | 3 (23.1%) | 0.088 | 17 (7.9%) | 2 (28.6%) | 0.113 | 14 (7.9%) | 5 (11.4%) | 0.546 | 19 (8.8%) | 0 (0.0%) | 1.000 |
| **BCR/ABL1** | 21 (10.0%) | 0 (0.0%) | 0.618 | 20 (9.3%) | 1 (14.3%) | 0.506 | 20 (11.2%) | 1 (2.3%) | 0.085 | 20 (9.3%) | 1 (14.3%) | 0.506 |
| **Risk Stratification** |  |  |  |  |  |  |  |  |  |  |  |  |
| Favorable | 65 (31.1%) | 6 (46.2%) | 0.467 | 70 (32.6%) | 1 (14.3%) | 0.567 | 61 (34.3%) | 10 (22.7%) | 0.002^*^ | 68 (31.6%) | 3 (42.9%) | 0.719 |
| Intermediate | 81 (38.8%) | 5 (38.5%) |  | 83 (38.6%) | 3 (42.9%) |  | 59 (33.1%) | 27 (61.4%) |  | 83 (38.6%) | 3 (42.9%) |  |
| Poor | 63 (30.1%) | 2 (15.4%) |  | 62 (28.8%) | 3 (42.9%) |  | 58 (32.6%) | 7 (15.9%) |  | 64 (29.8%) | 1 (14.3%) |  |
| **Response to induction** |  |  |  |  |  |  |  |  |  |  |  |  |
| CR | 94 (45.0%) | 4 (30.8%) | 0.284 | 93 (43.3%) | 5 (71.4%) | 0.731 | 82 (46.1%) | 16 (36.4%) | 0.324 | 95 (44.2%) | 3 (42.9%) | 0.714 |
| PR | 7 (3.3%) | 0 (0.0%) |  | 7 (3.3%) | 0 (0.0%) |  | 6 (3.4%) | 1 (2.3%) |  | 7 (3.3%) | 0 (0.0%) |  |
| Refractory | 35 (16.7%) | 4 (30.8%) |  | 39 (18.1%) | 0 (0.0%) |  | 33 (18.5%) | 6 (13.6%) |  | 37 (17.2%) | 2 (28.6%) |  |
| Induction death | 31 (14.8%) | 4 (30.8%) |  | 34 (15.8%) | 1 (14.3%) |  | 24 (13.5%) | 11 (25.0%) |  | 35 (16.3%) | 0 (0.0%) |  |
| Not applicable | 42 (20.1%) | 1 (7.7%) |  | 42 (19.5%) | 1 (14.3%) |  | 33 (18.5%) | 10 (22.7%) |  | 41 (19.1%) | 2 (28.6%) |  |
| **Relapse for CR** | 25/94(26.6%) | 1/4(25.0%) | 1.000 | 25/93(26.9%) | 1/5(20.0%) | 1.000 | 22/82(26.8%) | 4/16(25.0%) | 1.000 | 26/95(27.4%) | 0/3(0.0%) | 0.563 |
| **Response to salvage** |  |  |  |  |  |  |  |  |  |  |  |  |
| CR | 18 (8.6%) | 0 (0.0%) | 0.545 | 17 (7.9%) | 1 (14.3%) | 0.547 | 16 (9.0%) | 2 (4.5%) | 0.086 | 17 (7.9%) | 1 (14.3%) | 0.374 |
| PR | 20 (9.6%) | 2 (15.4%) |  | 22 (10.2%) | 0 (0.0%) |  | 21 (11.8%) | 1 (2.3%) |  | 21 (9.8%) | 1 (14.3%) |  |
| Not applicable | 171 (81.8%) | 11 (84.6%) |  | 176 (81.9%) | 6 (85.7%) |  | 141 (79.2%) | 41 (93.2%) |  | 177 (82.3%) | 5 (71.4%) |  |
| **CNS infiltration** | 24 (11.5%) | 4 (30.8%) | 0.065 | 28 (13.0%) | 0 (0.0%) | 0.600 | 27 (15.2%) | 1 (2.3%) | 0.021^*^ | 27 (12.6%) | 1 (14.3%) | 1.000 |

SD.: Standard deviation, SE.: Standard error, Min.: Minimum, Max.: Maximum, t: Student t-test, U: Mann–Whitney, X^2^: Chi-Square, FE: Fisher Exact, MC: Monte Carlo, P: Comparing the different categories, *: Significant.

***Supplementary Table 4. Association between optic disc infiltration, disc pallor, venous congestion and tortuosity, and retinal infiltration with different parameters***

|  | **Optic disc infiltration** | | | **Disc pallor** | | | **Venous congestion & tortuosity** | | | **Retinal infiltration** | | |
| --- | --- | --- | --- | --- | --- | --- | --- | --- | --- | --- | --- | --- |
|  | **Absent N = 218** | **Present N = 4** | **p** | **Absent N = 218** | **Present N = 4** | **p** | **Absent N = 213** | **Present N = 9** | **p** | **Absent N = 218** | **Present N = 4** | **p** |
| **Hb (g/dl)** |  |  |  |  |  |  |  |  |  |  |  |  |
| Mean ± SD. | 8.54 ± 2.04 | 7.08 ± 2.62 | 0.157 | 8.5 ± 2.04 | 9.25 ± 2.87 | 0.472 | 8.56 ± 2.04 | 7.57 ± 2.33 | 0.158 | 8.55 ± 2.05 | 6.85 ± 2.13 | 0.102 |
| Median (Min. – Max.) | 8.5(3 – 15) | 7.15(4 – 10) |  | 8.5(3 – 15) | 8.5(7 – 13) |  | 8.5(3 – 15) | 8.7(4 – 10.2) |  | 8.5(3 – 15) | 6.25(5 – 9.9) |  |
| **TLC (k/uL)** |  |  |  |  |  |  |  |  |  |  |  |  |
| Mean ± SE. | 55.5 ± 5.65 | 29.5 ± 14 | 1.000 | 55.3 ± 5.63 | 41.1 ± 35.1 | 0.509 | 54.9 ± 5.7 | 57.5 ± 25.4 | 0.935 | 52.6 ± 4.89 | 188 ± 161 | 0.352 |
| Median (Min. – Max.) | 19(0.3 – 670) | 26.6(4 – 61) |  | 19(0.3 – 670) | 8.9(0.4 – 146) |  | 19(0.3 – 670) | 45(0.4 – 237) |  | 18(0.3 – 449) | 37(6 – 670) |  |
| **Plt count (k/uL)** |  |  |  |  |  |  |  |  |  |  |  |  |
| Mean ± SE. | 58 ± 4.55 | 43.2 ± 17.4 | 0.997 | 57.2 ± 4.52 | 86.8 ± 32.5 | 0.173 | 58 ± 4.64 | 52 ± 12.4 | 0.375 | 58.3 ± 4.55 | 30.8 ± 11.8 | 0.346 |
| Median (Min. – Max.) | 32(2 – 512) | 32(15.9 – 94) |  | 32(2 – 512) | 80(26 – 161) |  | 32(2 – 512) | 40(15 – 141) |  | 32(2 – 512) | 29(10 – 55) |  |
| **Blast % in PB** |  |  |  |  |  |  |  |  |  |  |  |  |
| Mean ± SE. | 60.3 ± 1.75 | 71.3 ± 12.97 | 0.331 | 60.3 ± 1.74 | 73 ± 14.3 | 0.241 | 60 ± 1.77 | 73.8 ± 7.42 | 0.108 | 60.3 ± 1.75 | 70.8 ± 12.6 | 0.440 |
| Median (Min. – Max.) | 60(5 – 98) | 75(40 – 95) |  | 60(5 – 98) | 82.5(32 – 95) |  | 60(5 – 98) | 78(27 – 95) |  | 60(5 – 98) | 79(35 – 90) |  |
| **Blast % in BM** |  |  |  |  |  |  |  |  |  |  |  |  |
| Mean ± SD. | 74.9 ± 22 | 73.3 ± 25.9 | 0.886 | 75.1 ± 21.7 | 61 ± 34.7 | 0.205 | 74.7 ± 22 | 77.7 ± 21.8 | 0.693 | 74.6 ± 22.1 | 87.8 ± 5.19 | 0.006* |
| Median (Min. – Max.) | 85(6 – 98) | 80(38 – 95) |  | 85(6 – 98) | 61(30 – 92) |  | 85(6 – 98) | 90(30 – 95) |  | 85(6 – 98) | 90(80 – 91) |  |
| **Cytogenetics** | 63 (28.9%) | 1 (25.0%) | 1.000 | 63 (28.9%) | 1 (25.0%) | 1.000 | 63 (29.6%) | 1 (11.1%) | 0.452 | 62 (28.4%) | 2 (50.0%) | 0.581 |
| **Molecular abnormality** | 19 (8.7%) | 0 (0.0%) | 1.000 | 19 (8.7%) | 0 (0.0%) | 1.000 | 19 (8.9%) | 0 (0.0%) | 1.000 | 19 (8.7%) | 0 (0.0%) | 1.000 |
| **BCR/ABL1** | 21 (9.6%) | 0 (0.0%) | 1.000 | 21 (9.6%) | 0 (0.0%) | 1.000 | 20 (9.4%) | 1 (11.1%) | 0.598 | 21 (9.6%) | 0 (0.0%) | 1.000 |
| **Risk Stratification** |  |  |  |  |  |  |  |  |  |  |  |  |
| Favorable | 70 (32.1%) | 1 (25.0%) | 1.000 | 70 (32.1%) | 1 (25.0%) | 0.387 | 69 (32.4%) | 2 (22.2%) | 0.261 | 69 (31.7%) | 2 (50.0%) | 0.827 |
| Intermediate | 84 (38.5%) | 2 (50.0%) |  | 83 (38.1%) | 3 (75.0%) |  | 80 (37.6%) | 6 (66.7%) |  | 85 (39.0%) | 1 (25.0%) |  |
| Poor | 64 (29.4%) | 1 (25.0%) |  | 65 (29.8%) | 0 (0.0%) |  | 64 (30.0%) | 1 (11.1%) |  | 64 (29.4%) | 1 (25.0%) |  |
| **Response to induction** |  |  |  |  |  |  |  |  |  |  |  |  |
| CR | 97 (44.5%) | 1 (25.0%) | 0.388 | 97 (44.5%) | 1 (25.0%) | 0.271 | 91 (42.7%) | 7 (77.8%) | 0.274 | 95 (43.6%) | 3 (75.0%) | 0.709 |
| PR | 7 (3.2%) | 0 (0.0%) |  | 7 (3.2%) | 0 (0.0%) |  | 7 (3.3%) | 0 (0.0%) |  | 7 (3.2%) | 0 (0.0%) |  |
| Refractory | 37 (17.0%) | 2 (50.0%) |  | 39 (17.9%) | 0 (0.0%) |  | 39 (18.3%) | 0 (0.0%) |  | 38 (17.4%) | 1 (25.0%) |  |
| Induction death | 35 (16.1%) | 0 (0.0%) |  | 33 (15.1%) | 2 (50.0%) |  | 35 (16.4%) | 0 (0.0%) |  | 35 (16.1%) | 0 (0.0%) |  |
| Not applicable | 42 (19.3%) | 1 (25.0%) |  | 42 (19.3%) | 1 (25.0%) |  | 41 (19.2%) | 2 (22.2%) |  | 43 (19.7%) | 0 (0.0%) |  |
| **Relapse for CR** | 26/97(26.8%) | 0/1(0.0%) | 1.000 | 26/97(26.8%) | 0/1(0.0%) | 1.000 | 26/91(28.6%) | 0/7(0.0%) | 0.184 | 24/95(25.3%) | 2/3(66.7%) | 0.171 |
| **Response to salvage** |  |  |  |  |  |  |  |  |  |  |  |  |
| CR | 18 (8.3%) | 0 (0.0%) | 1.000 | 18 (8.3%) | 0 (0.0%) | 1.000 | 18 (8.5%) | 0 (0.0%) | 0.813 | 17 (7.8%) | 1 (25.0%) | 0.148 |
| PR | 22 (10.1%) | 0 (0.0%) |  | 22 (10.1%) | 0 (0.0%) |  | 22 (10.3%) | 0 (0.0%) |  | 21 (9.6%) | 1 (25.0%) |  |
| Not applicable | 178 (81.7%) | 4 (100.0%) |  | 178 (81.7%) | 4 (100.0%) |  | 173 (81.2%) | 9 (100.0%) |  | 180 (82.6%) | 2 (50.0%) |  |
| **CNS infiltration** | 28 (12.8%) | 0 (0.0%) | 1.000 | 27 (12.4%) | 1 (25.0%) | 0.419 | 28 (13.1%) | 0 (0.0%) | 0.607 | 27 (12.4%) | 1 (25.0%) | 0.419 |

SD.: Standard deviation, SE.: Standard error, Min.: Minimum, Max.: Maximum, t: Student t-test, U: Mann–Whitney, X^2^: Chi-Square, FE: Fisher Exact, MC: Monte Carlo, P: Comparing the different categories, *: Significant.

***Supplementary Table 5. Association between exudative retinal detachment, ocular motility, orbital involvement, and macula affection with hematological parameters at diagnosis.***

|  | **Exudative retinal detachment** | | | **Ocular motility** | | | **Orbital involvement** | | | **Macula affection** | | |
| --- | --- | --- | --- | --- | --- | --- | --- | --- | --- | --- | --- | --- |
|  | **Absent N = 218** | **Present N = 4** | **p** | **Absent N = 219** | **Present N = 3** | **p** | **Absent N = 215** | **Present N = 7** | **p** | **Absent N = 217** | **Present N = 5** | **p** |
| **Hb (g/dl)** |  |  |  |  |  |  |  |  |  |  |  |  |
| Mean ± SD. | 8.53 ± 2.06 | 7.48 ± 1.67 | 0.308 | 8.52 ± 2.06 | 8.50 ± 1.50 | 0.989 | 8.52 ± 2.05 | 8.31 ± 2.35 | 0.793 | 8.52 ± 2.06 | 8.20 ± 2.04 | 0.729 |
| Median (Min. – Max.) | 8.5(3 – 15) | 8.15(5 – 8.6) |  | 8.5(3 – 15) | 8.5(7 – 10) |  | 8.5(3 – 15) | 8(5 – 11.7) |  | 8.5(3 – 15) | 8.5(5.4 –10.5) |  |
| **TLC (k/uL)** |  |  |  |  |  |  |  |  |  |  |  |  |
| Mean ± SE. | 54.7 ± 5.61 | 72.5 ± 44.2 | 0.512 | 54.7 ± 5.56 | 81.4 ± 79.3 | 0.529 | 55 ± 5.65 | 55.5 ± 33.6 | 0.830 | 54.3 ± 5.61 | 89.3 ± 39.6 | 0.297 |
| Median (Min. – Max.) | 19(0.3 – 670) | 45.1(6 – 194) |  | 19(0.3 – 670) | 3.9(0.4 – 240) |  | 19(0.3 – 670) | 10(0.4 – 240) |  | 18(0.3 – 670) | 48(1.3 – 215) |  |
| **Plt count (k/uL)** |  |  |  |  |  |  |  |  |  |  |  |  |
| Mean ± SE. | 58.5 ± 4.54 | 18.8 ± 3.51 | 0.087 | 56.9 ± 4.37 | 125 ± 99 | 0.690 | 56.4 ± 4.41 | 99.6 ± 43 | 0.316 | 57.9 ± 4.56 | 50.9 ± 18.8 | 0.751 |
| Median (Min. – Max.) | 33(2 – 512) | 19(11 – 27.4) |  | 32(2 – 512) | 39(13 – 322) |  | 32(2 – 512) | 39(11 – 322) |  | 32(2 – 512) | 26(22 – 121) |  |
| **Blast % in PB** |  |  |  |  |  |  |  |  |  |  |  |  |
| Mean ± SE. | 60.5 ± 1.75 | 62.8 ± 11.3 | 0.884 | 60.3 ± 1.74 | 76 ± 14.7 | 0.282 | 60.4 ± 1.74 | 64.6 ± 13.3 | 0.479 | 60.3 ± 1.75 | 69.4 ± 11.3 | 0.393 |
| Median (Min. – Max.) | 60(5 – 98) | 63(35 – 90) |  | 60(5 – 98) | 86(47 – 95) |  | 60(5 – 98) | 89(6 – 95) |  | 60(5 – 98) | 75(37 – 95) |  |
| **Blast % in BM** |  |  |  |  |  |  |  |  |  |  |  |  |
| Mean ± SD. | 74.6 ± 22.1 | 87.3 ± 3.86 | 0.001^*^ | 75 ± 21.9 | 60 ± 30 | 0.241 | 74.9 ± 22 | 73 ± 23.7 | 0.825 | 74.6 ± 22.2 | 85.2 ± 10.1 | 0.287 |
| Median (Min. – Max.) | 85(6 – 98) | 87.5(83 – 91) |  | 85(6 – 98) | 60(30 – 90) |  | 85(6 – 98) | 90(30 – 91) |  | 85(6 – 98) | 90(72 – 95) |  |
| **Cytogenetics** | 62 (28.4%) | 2 (50.0%) | 0.581 | 63 (28.8%) | 1 (33.3%) | 1.000 | 61 (28.4%) | 3 (42.9%) | 0.414 | 61 (28.1%) | 3 (60.0%) | 0.145 |
| **Molecular abnormality** | 19 (8.7%) | 0 (0.0%) | 1.000 | 19 (8.7%) | 0 (0.0%) | 1.000 | 18 (8.4%) | 1 (14.3%) | 0.470 | 19 (8.8%) | 0 (0.0%) | 1.000 |
| **BCR/ABL1** | 21 (9.6%) | 0 (0.0%) | 1.000 | 20 (9.1%) | 1 (33.3%) | 0.259 | 20 (9.3%) | 1 (14.3%) | 0.506 | 21 (9.7%) | 0 (0.0%) | 1.000 |
| **Risk Stratification** |  |  |  |  |  |  |  |  |  |  |  |  |
| Favorable | 69 (31.7%) | 2 (50.0%) | 0.557 | 70 (32.0%) | 1 (33.3%) | 1.000 | 70 (32.6%) | 1 (14.3%) | 0.341 | 68 (31.3%) | 3 (60.0%) | 0.275 |
| Intermediate | 84 (38.5%) | 2 (50.0%) |  | 85 (38.8%) | 1 (33.3%) |  | 84 (39.1%) | 2 (28.6%) |  | 84 (38.7%) | 2 (40.0%) |  |
| Poor | 65 (29.8%) | 0 (0.0%) |  | 64 (29.2%) | 1 (33.3%) |  | 61 (28.4%) | 4 (57.1%) |  | 65 (30.0%) | 0 (0.0%) |  |
| **Response to induction** |  |  |  |  |  |  |  |  |  |  |  |  |
| CR | 97 (44.5%) | 1 (25.0%) | 0.426 | 97 (44.3%) | 1 (33.3%) | 0.364 | 97 (45.1%) | 1 (14.3%) | 0.082 | 96 (44.2%) | 2 (40.0%) | 0.261 |
| PR | 7 (3.2%) | 0 (0.0%) |  | 7 (3.2%) | 0 (0.0%) |  | 7 (3.3%) | 0 (0.0%) |  | 6 (2.8%) | 1 (20.0%) |  |
| Refractory | 38 (17.4%) | 1 (25.0%) |  | 39 (17.8%) | 0 (0.0%) |  | 36 (16.7%) | 3 (42.9%) |  | 39 (18.0%) | 0 (0.0%) |  |
| Induction death | 35 (16.1%) | 0 (0.0%) |  | 35 (16.0%) | 0 (0.0%) |  | 35 (16.3%) | 0 (0.0%) |  | 34 (15.7%) | 1 (20.0%) |  |
| Not applicable | 41 (18.8%) | 2 (50.0%) |  | 41 (18.7%) | 2 (66.7%) |  | 40 (18.6%) | 3 (42.9%) |  | 42 (19.4%) | 1 (20.0%) |  |
| **Relapse for CR** | 26/97(26.8%) | 0/1(0.0%) | 1.000 | 26/97(26.8%) | 0/1(0.0%) | 1.000 | 26/97(26.8%) | 0/1(0.0%) | 1.000 | 26/96(27.1%) | 0/2(0.0%) | 1.000 |
| **Response to salvage** |  |  |  |  |  |  |  |  |  |  |  |  |
| CR | 18 (8.3%) | 0 (0.0%) | 0.545 | 18 (8.2%) | 0 (0.0%) | 1.000 | 18 (8.4%) | 0 (0.0%) | 0.235 | 18 (8.3%) | 0 (0.0%) | 1.000 |
| PR | 21 (9.6%) | 1 (25.0%) |  | 22 (10.0%) | 0 (0.0%) |  | 20 (9.3%) | 2 (28.6%) |  | 22 (10.1%) | 0 (0.0%) |  |
| Not applicable | 179 (82.1%) | 3 (75.0%) |  | 179 (81.7%) | 3 (100.0%) |  | 177 (82.3%) | 5 (71.4%) |  | 177 (81.6%) | 5 (100.0%) |  |
| **CNS infiltration** | 28 (12.8%) | 0 (0.0%) | 1.000 | 28 (12.8%) | 0 (0.0%) | 1.000 | 26 (12.1%) | 2 (28.6%) | 0.216 | 28 (12.9%) | 0 (0.0%) | 1.000 |

SD.: Standard deviation, SE.: Standard error, Min.: Minimum, Max.: Maximum, t: Student t-test, U: Mann–Whitney, X^2^: Chi-Square, FE: Fisher Exact, MC: Monte Carlo, P: Comparing the different categories, *: Significant.

***Supplementary Table 6. Association between conjunctival chemosis, Roth spots, cotton wool spots, and papilledema with different parameters***

|  | **Conjunctival chemosis** | | | **Roth spots** | | | **Cotton wool spots** | | | **Papilledema** | | |
| --- | --- | --- | --- | --- | --- | --- | --- | --- | --- | --- | --- | --- |
|  | **Absent N = 220** | **Present N = 2** | **p** | **Absent N = 184** | **Present N = 38** | **p** | **Absent N = 220** | **Present N = 2** | **p** | **Absent N = 216** | **Present N = 6** | **p** |
| **Hb (g/dl)** |  |  |  |  |  |  |  |  |  |  |  |  |
| Mean ± SD. | 8.53 ± 2.06 | 7.50 ± 0.71 | 0.484 | 8.61 ± 2.08 | 8.07 ± 1.90 | 0.141 | 8.52 ± 2.05 | 8 ± 2.83 | 0.722 | 8.47 ± 2.04 | 10.07 ± 2.32 | 0.061 |
| Median (Min. – Max.) | 8.50(3 – 15) | 7.50(7 – 8) |  | 8.50(3 – 15) | 8.35(4 – 12) |  | 8.50(3 – 15) | 8(6 – 10) |  | 8.40(3 – 15) | 9.45(7 – 13) |  |
| **TLC (k/uL)** |  |  |  |  |  |  |  |  |  |  |  |  |
| Mean ± SE. | 55.5 ± 5.6 | 3.2 ± 2.8 | 0.094 | 54 ± 6.3 | 60.2 ± 10.6 | 0.098 | 55.5 ± 5.6 | 2.6 ± 1 | 0.082 | 55.7 ± 5.68 | 31 ± 22 | 0.161 |
| Median (Min. – Max.) | 19(0.3 – 670) | 3.2(0.4 – 6) |  | 17(0.3 – 670) | 39(0.4 – 300) |  | 19(0.3 – 670) | 2.6(1.6 – 3.6) |  | 19(0.3 – 67) | 4.1(0.9 – 137) |  |
| **Plt count (k/uL)** |  |  |  |  |  |  |  |  |  |  |  |  |
| Mean ± SE. | 57.7 ± 4.51 | 64.5 ± 25.5 | 0.353 | 59.9 ± 5.14 | 47.3 ± 7.91 | 0.680 | 57.6 ± 4.51 | 76 ± 47 | 0.432 | 58.4 ± 4.58 | 35.2 ± 12 | 0.408 |
| Median (Min. – Max.) | 32(2 – 512) | 64.5(39 – 90) |  | 33(2 – 512) | 29.5(7.2–232) |  | 32(2 – 512) | 76(29 – 123) |  | 32.5(2 – 512) | 28.7(9 – 88) |  |
| **Blast % in PB** |  |  |  |  |  |  |  |  |  |  |  |  |
| Mean ± SE. | 60.6 ± 1.72 | 50.5 ± 44.5 | 0.960 | 59 ± 1.9 | 68 ± 3.98 | 0.051 | 60.7 ± 1.74 | 40 ± 20 | 0.256 | 60.3 ± 1.76 | 70.2 ± 10.3 | 0.315 |
| Median (Min. – Max.) | 60(5 – 98) | 50.50(6 – 95) |  | 60(6 – 98) | 71.50(5 – 95) |  | 61(5 – 98) | 40(20 – 60) |  | 60(5 – 98) | 80.5(30 – 90) |  |
| **Blast % in BM** |  |  |  |  |  |  |  |  |  |  |  |  |
| Mean ± SD. | 75.1 ± 21.9 | 45 ± 21.2 | 0.054 | 75.2 ± 21.7 | 73 ± 23.5 | 0.582 | 75 ± 21.9 | 53.5 ± 26.2 | 0.169 | 74.7 ± 22 | 80 ± 24.5 | 0.560 |
| Median (Min. – Max.) | 85(6 – 98) | 45(30 – 60) |  | 85(13 – 98) | 81.5(6 – 95) |  | 85(6 – 98) | 53.5(35 – 72) |  | 85(6 – 98) | 90(30 – 90) |  |
| **Cytogenetics** | 64 (29.1%) | 0 (0.0%) | 1.000 | 54 (29.3%) | 10 (26.3%) | 0.707 | 64 (29.1%) | 0 (0.0%) | 1.000 | 61 (28.2%) | 3 (50.0%) | 0.358 |
| **Molecular abnormality** | 19 (8.6%) | 0 (0.0%) | 1.000 | 13 (7.1%) | 6 (15.8%) | 0.106 | 19 (8.6%) | 0 (0.0%) | 1.000 | 19 (8.8%) | 0 (0.0%) | 1.000 |
| **BCR/ABL1** | 21 (9.5%) | 0 (0.0%) | 1.000 | 20 (10.9%) | 1 (2.6%) | 0.138 | 21 (9.5%) | 0 (0.0%) | 1.000 | 21 (9.7%) | 0 (0.0%) | 1.000 |
| **Risk Stratification** |  |  |  |  |  |  |  |  |  |  |  |  |
| Favorable | 71 (32.3%) | 0 (0.0%) | 0.754 | 62 (33.7%) | 9 (23.7%) | 0.269 | 71 (32.3%) | 0 (0.0%) | 0.332 | 67 (31.0%) | 4 (66.7%) | 0.283 |
| Intermediate | 85 (38.6%) | 1 (50.0%) |  | 67 (36.4%) | 19 (50.0%) |  | 84 (38.2%) | 2 (100.0%) |  | 85 (39.4%) | 1 (16.7%) |  |
| Poor | 64 (29.1%) | 1 (50.0%) |  | 55 (29.9%) | 10 (26.3%) |  | 65 (29.5%) | 0 (0.0%) |  | 64 (29.6%) | 1 (16.7%) |  |
| **Response to induction** |  |  |  |  |  |  |  |  |  |  |  |  |
| CR | 98 (44.5%) | 0 (0.0%) | 0.340 | 78 (42.4%) | 20 (52.6%) | 0.472 | 97 (44.1%) | 1 (50.0%) | 0.479 | 93 (43.1%) | 5 (83.3%) | 0.455 |
| PR | 7 (3.2%) | 0 (0.0%) |  | 6 (3.3%) | 1 (2.6%) |  | 7 (3.2%) | 0 (0.0%) |  | 7 (3.2%) | 0 (0.0%) |  |
| Refractory | 38 (17.3%) | 1 (50.0%) |  | 34 (18.5%) | 5 (13.2%) |  | 39 (17.7%) | 0 (0.0%) |  | 39 (18.1%) | 0 (0.0%) |  |
| Induction death | 35 (15.9%) | 0 (0.0%) |  | 32 (17.4%) | 3 (7.9%) |  | 34 (15.5%) | 1 (50.0%) |  | 34 (15.7%) | 1 (16.7%) |  |
| Not applicable | 42 (19.1%) | 1 (50.0%) |  | 34 (18.5%) | 9 (23.7%) |  | 43 (19.5%) | 0 (0.0%) |  | 43 (19.9%) | 0 (0.0%) |  |
| **Relapse for CR** | 26/98(26.5%) | – | – | 22/78(28.2%) | 4/20(20.0%) | 0.458 | 26/97(26.8%) | 0/1(0.0%) | 1.000 | 25/93(26.9%) | 1/5(20.0%) | 1.000 |
| **Response to salvage** |  |  |  |  |  |  |  |  |  |  |  |  |
| CR | 18 (8.2%) | 0 (0.0%) | 1.000 | 18 (9.8%) | 0 (0.0%) | 0.075 | 18 (8.2%) | 0 (0.0%) | 1.000 | 17 (7.9%) | 1 (16.7%) | 0.476 |
| PR | 22 (10.0%) | 0 (0.0%) |  | 17 (9.2%) | 5 (13.2%) |  | 22 (10.0%) | 0 (0.0%) |  | 22 (10.2%) | 0 (0.0%) |  |
| Not applicable | 180 (81.8%) | 2 (100.0%) |  | 149 (81.0%) | 33 (86.8%) |  | 180 (81.8%) | 2 (100.0%) |  | 177 (81.9%) | 5 (83.3%) |  |
| **CNS infiltration** | 28 (12.7%) | 0 (0.0%) | 1.000 | 23 (12.5%) | 5 (13.2%) | 1.000 | 28 (12.7%) | 0 (0.0%) | 1.000 | 26 (12.0%) | 2 (33.3%) | 0.167 |

SD.: Standard deviation, SE.: Standard error, Min.: Minimum, Max.: Maximum, t: Student t-test, U: Mann–Whitney, X^2^: Chi-Square, FE: Fisher Exact, MC: Monte Carlo, P: Comparing the different categories, *: Significant.
